# Advancement in piezoelectric nanogenerators for acoustic energy harvesting

**DOI:** 10.1038/s41378-024-00811-4

**Published:** 2024-12-18

**Authors:** Fandi Jean, Muhammad Umair Khan, Anas Alazzam, Baker Mohammad

**Affiliations:** 1 Department of Computer and Information Engineering, Khalifa University, Abu Dhabi, 12778 UAE; 2 System on Chip Lab, Khalifa University, Abu Dhabi, 12778 UAE; 3 Department of Mechanical Engineering, Khalifa University, Abu Dhabi, 12778 UAE

**Keywords:** Sensors, Electrical and electronic engineering, Electronic devices

## Abstract

The demand for sustainable energy sources to power small electronics like IoT devices has led to exploring innovative solutions like acoustic energy harvesting using piezoelectric nanogenerators (PENGs). Acoustic energy harvesting leverages ambient noise, converting it into electrical energy through the piezoelectric effect, where certain materials generate an electric charge in response to mechanical stress or vibrations. This review paper provides a comprehensive analysis of the advancements in PENG technology, emphasizing their role in acoustic energy harvesting. We begin by discussing the essential principles of piezoelectricity and the design considerations for nanogenerators to optimize energy capture from sound waves. The discussion includes a detailed examination of various piezoelectric materials, such as polyvinylidene fluoride (PVDF), lead zirconate titanate (PZT), and zinc oxide (ZnO) nanowires, which are known for their superior piezoelectric properties. A critical aspect of this review is the exploration of innovative structural designs and resonance devices that enhance the efficiency of PENGs. We delve into the mechanisms and benefits of using Helmholtz resonators, quarter-wavelength tubes, and cantilever beams, which are instrumental in amplifying acoustic signals and improving energy conversion rates. Each device’s design parameters and operational principles are scrutinized to highlight their contributions to the field. The review addresses practical applications of PENGs in various domains. Environmental monitoring systems, wearable electronics, and medical devices stand to benefit significantly from the continuous and sustainable power supplied by PENGs. These applications can reduce reliance on batteries and minimize maintenance by harnessing ambient acoustic energy, leading to more efficient and longer-lasting operations. Despite the promising potential of PENGs, several challenges remain, including material degradation, efficiency limitations, and integrating these devices into existing technological frameworks. This paper discusses these obstacles in detail and proposes potential solutions to enhance the longevity and performance of PENG systems. Innovations in material science and engineering are crucial to overcoming these hurdles and realizing the full potential of acoustic energy harvesting.

## Introduction

Capturing and converting ambient energy from the environment for future use, known as energy harvesting, has become increasingly prominent in pursuing green and renewable energy solutions^1^. The urgent challenge of mitigating climate change, reducing greenhouse gas emissions, and moving away from reliance on fossil fuels has motivated a broad spectrum of researchers, innovators, and technology developers to explore alternative methods of generating power^2–4^ Energy harvesting stands out as a critical strategy in this effort, offering multiple benefits^5,6^. As the proliferation of electronic devices escalates global energy use, addressing this surge in demand becomes critical^2,7^. Energy harvesting introduces an innovative approach by tapping underutilized environmental energy sources^8,9^. Thus contributing to meeting the increasing need for power without overburdening current energy systems. Furthermore, energy harvesting is crucial in remote and underserved areas, where access to consistent and reliable energy sources is scarce^10,11^. This issue is particularly acute in many developing regions that lack comprehensive power infrastructure, making energy access a significant hurdle^12,13^. Energy harvesting technology empowers these communities by providing a localized, independent energy solution. In the era of the Internet of Things (IoT), the relevance of energy harvesting has become even more pronounced^1,14^. IoT applications, from consumer electronics to sophisticated industrial monitors, depend on uninterrupted power supplies^14–16^. Given the logistical challenges of battery replacement or conventional energy provisioning in inaccessible areas, energy harvesters offer a sustainable, self-replenishing energy source to these devices, enhancing their efficiency and reducing upkeep^15–18^. This synergy between IoT and energy harvesting promises to expand IoT networks, improving data gathering, analysis, and automation across numerous industries^19–21^. Energy harvesting methods innovate by transforming readily available environmental energies such as electromagnetic, thermal, and solar- into electric power^22,23^. These techniques exploit sources like radiofrequency waves from electronic devices, ambient thermal differences, or sunlight via photovoltaic cells, presenting a sustainable strategy to augment existing energy supplies, lessen fossil fuel dependency, and cater to the rising energy demands sustainably^24–30^. Moreover, mechanical energy harvesting emerges as a versatile solution, converting the kinetic energy from physical movements and vibrations into electricity^31,32^. Unlike conventional harvesting methods that require more substantial, consistent environmental inputs, piezoelectric nanogenerators (PENGs) thrive on low-frequency, variable mechanical inputs^31^, making them suitable for various settings and uses. Their compact, lightweight design allows easy integration into small-scale electronics and wearables, offering adaptability and scalability for everything from individual sensors to contributions to the broader power grid^33,34^. The piezoelectric effect refers to the capacity of specific materials to produce electrical charges in response to the application of external mechanical energy. The aforementioned phenomenon exhibits reversibility, whereby the application of an electric field can induce a corresponding deformation in the material. Piezoelectric materials are utilized in diverse applications, such as sensors, actuators, transducers, and energy harvesting devices^35^. In addition to energy harvesting, signal and data processing in PENGs are critical for optimizing their performance and applications. These processes involve converting the generated electrical signals from mechanical energy into a usable form, typically by rectifying and filtering the output to produce a stable direct current (DC). This conversion is essential for powering electronic devices and ensuring consistent energy delivery. Advanced signal processing techniques also allow for the analysis and interpretation of the electrical signals generated by PENGs, enabling real-time monitoring of environmental conditions and device performance. Data processing algorithms can further enhance the functionality of PENGs by managing energy storage, distribution, and consumption efficiently. This combination of energy harvesting with sophisticated signal and data processing not only improves the reliability and efficiency of PENGs but also expands their potential applications in areas such as healthcare, environmental monitoring, and smart infrastructure^36,37^.

PENGs can exploit various forms of mechanical energies, ranging from evident actions like squeezing and flexing to more subtle dynamics such as changes due to thermal effects or slight vibrations from liquid movements. Each category of mechanical force is uniquely suited to specific scenarios and applications, yet also comes with its own challenges. Acoustic energy, in particular, emerges as a notable source among these. This method harnesses the vibrational energy present in sound waves, converting it into electrical power^38^. Our environment is replete with sounds: the gentle flutter of leaves, the unyielding drone of urban life, and the diverse voices of human interaction. This constant sound environment offers a dependable and adaptable energy source^39^. Converting ubiquitous sound into a renewable energy source makes everyday noises valuable for powering self-sustaining devices. This opens transformative possibilities for how devices could function autonomously. Imagine environmental sensors that leverage ambient sound to continuously monitor factors such as air pollution, noise levels, or thermal conditions, or wearable technology like fitness monitors and smartwatches^40^ that could draw energy from environmental noise or the wearer’s own speech, ensuring they never run out of power. The endless nature of acoustic energy points towards a future enriched with more sustainable technological advancements. Additionally, leveraging acoustic energy offers a creative solution to the issue of noise pollution, which is especially prevalent in urban landscapes. By converting commonly regarded disruptive urban noise into a valuable energy resource, this approach not only provides an ingenious solution to urban noise pollution but also encourages the shift towards renewable energy practices, marking a significant leap in efforts to protect the environment^41,42^.

To better understand the concept of acoustic energy harvesting, it’s crucial to first grasp what sound waves are. At their core, sound waves are mechanical vibrations that travel through a medium, such as air, and are the primary means of communication for humans and various other species. These waves originate from the oscillation or movement of objects, which then cause the air around them to vibrate. This initial vibration prompts nearby air molecules to oscillate, creating a domino effect that propagates the wave through the medium. The energy a sound wave carries, which determines its volume or intensity, is directly linked to its amplitude, with the decibel (dB) serving as the logarithmic scale for measuring this intensity. Frequency, measured in hertz (Hz), describes how often the sound wave cycles occur within a second, influencing the perceived pitch of the sound — its perceived highness or lowness. Human hearing spans frequencies from about 20 Hz to 20,000 Hz, with the lower end associated with deeper tones and the higher end with sharper sounds like bird calls or a telephone ringing. When considering the use of acoustic energy for consistent power, the challenges are multifaceted, mainly due to the variable nature of sound waves. Their intensity and frequency, pivotal for their energy-carrying capacity, vary significantly depending on numerous environmental factors. This variability challenges ensuring a steady energy supply, particularly in fluctuating environments like cities. For instance, an urban area’s soundscape is rich for harvesting during busy periods but becomes sparse in quieter times, leading to unpredictable energy availability. Sound’s omnidirectional propagation requires harvesters to efficiently capture waves from various directions, a feature that current technologies, often designed for unidirectional capture, frequently miss. This becomes a significant limitation in environments with numerous and varied sound sources. The power density of sound waves is generally lower than that of other renewable energy sources such as solar or wind, positioning acoustic energy more as a supplementary rather than a primary power solution. Consequently, its applications are usually geared towards smaller-scale devices with lower energy requirements, such as remote sensors, wireless devices, or certain medical implants. These devices benefit from acoustic energy in scenarios where conventional power sources are unfeasible or the energy needs are minimal, highlighting the specialized role of acoustic energy in overcoming specific power challenges^38^.

Research and development efforts in acoustic energy harvesting are intensifying to address existing limitations and enhance the overall efficiency of energy capture. A key focus is improving nanogenerators’ performance, pivotal in converting sound wave vibrations into electrical energy. By optimizing these nanogenerators, a more significant amount of energy can be harnessed from the ambient sound. Nanogenerators detect the vibrations caused by sound waves and convert these motions into electrical signals. An important avenue of exploration involves the innovation of new materials and design approaches that enhance the ability of these devices to capture and convert sound energy more effectively. An exciting development in this area is the exploration of triboelectric materials. Given their ability to generate electricity from even slight vibrations, their high sensitivity to minimal mechanical disturbances positions them as prime candidates for acoustic energy harvesting. Design considerations for acoustic nanogenerators are manifold, with the frequency range being a critical aspect. Each nanogenerator is optimized for a specific range of frequencies within which it operates most efficiently. Performance tends to decline sharply outside this optimal range. Typically, these devices are more effective at lower frequencies, usually within the 100–500 Hz spectrum. The rapid separation of contact points at higher frequencies can result in inadequate charge transfer, reducing energy capture efficiency. The physical size of the nanogenerator also plays a vital role in the quantity of energy it can harvest. Devices with larger surface areas can intercept more sound waves, enhancing their power output. However, scaling up the size of these devices introduces challenges related to portability and the ease of integrating them with other technologies. Balancing the trade-offs between size and efficiency is a critical challenge in the ongoing development of acoustic energy harvesting technologies.

In acoustic energy harvesting, the efficiency of converting acoustic energy into electrical signals is of utmost importance, especially considering the inherently low energy levels typically available from sound. The ability of an acoustic energy harvesting system to maximize the conversion of available sound energy into electricity is crucial for enhancing its overall efficacy. The system’s sensitivity to sound waves is an essential determinant of its efficiency, influencing its ability to convert even low-level sound vibrations into significant electrical outputs effectively. High-sensitivity systems are particularly adept at transforming faint sound waves into measurable electrical energy, an advantage in settings where ambient noise is minimal. This sensitivity is critical for maintaining steady voltage outputs, ensuring the system’s consistent performance despite the sound intensity and frequency variations. While employing more powerful speakers might appear to increase production, such results should be interpreted cautiously due to the potential for artificially high input signals. Thus, optimizing the device’s sensitivity and efficiency is essential for achieving superior performance and producing significant outputs from minimal inputs. The research underscores the importance of sensitivity and efficiency, indicating that systems designed with these priorities excel in capturing and converting acoustic energy, thereby boosting efficiency.

Moreover, the efficiency of an acoustic nanogenerator is closely linked to its membrane’s properties. The membrane is vital in transforming sound vibrations into electrical energy, with its design significantly influencing the system’s overall efficiency. For example, choosing a membrane with a fibrous structure can enhance the device’s interaction with sound waves. Such a structure, often created through techniques like electrospinning, uses electric fields to weave polymer solutions into intricate fibrous patterns. This increases the membrane’s surface area, optimizing it for better sound absorption and conversion to mechanical vibrations. The choice of materials for the nanogenerator’s electrodes is also crucial, with a preference for those offering high conductivity and minimal defects. This ensures the efficient conveyance of generated electrical energy to the intended load, highlighting the importance of material selection in maximizing the efficiency of acoustic energy harvesting systems^43–45^. Resonance devices, such as Helmholtz resonator (HR) and quarter-wavelength resonator, stand out as excellent candidates for acoustic energy harvesting because they facilitate a greater extraction of electrical output than the initial yield. This improvement is primarily due to the amplification of the signal entering the system, thereby increasing its efficiency.

Helmholtz resonance refers to the phenomenon of air resonance in a cavity. This resonance type occurs when air is forced into a cavity, causing the air inside to vibrate at a specific natural frequency. The principle is widely observable in everyday life, notably when blowing across the top of a bottle, resulting in a resonant tone. Helmholtz resonance is fundamental in various fields, including acoustics, engineering, and physics. The resonator, termed an HR, consists of two key components: a cavity and a neck. The size and shape of these components are crucial in determining the resonant frequency, which is the frequency at which the system naturally oscillates. In the context of acoustics, Helmholtz resonance is instrumental in the design and analysis of musical instruments, architectural acoustics, and sound engineering. It is also used in automotive engineering to reduce noise and design exhaust systems. The underlying principle involves the air mass’s vibration in the resonator’s neck, acting analogously to a mass on a spring. When external forces, such as airflow, disturb this air mass, it oscillates and causes the air within the cavity to resonate. This phenomenon is characterized by its sharp and high-amplitude resonance curve, making it distinct from other types of acoustic resonance. The efficiency of the resonator is illustrated through the following Eq. (1).

1 $${Fr}=\frac{c}{2\pi }\sqrt{\frac{A}{V\times L}}$$

In this equation, “*Fr*” is the resonant frequency, “*c*” indicates the speed of sound, “*A*” is the cross-sectional area of the neck or aperture, “*V*” is the volume of the resonator’s cavity, and “*L*” denotes the effective neck length. The selection of each geometric factor in the HR must be carefully executed to guarantee optimal functioning. The resonator’s effectiveness and specific resonant frequency depend heavily on these dimensions. Accurate adjustment and thoughtful analysis of these factors are crucial for obtaining the intended operational performance and resonance features tailored to particular uses^46^.

A quarter-wavelength resonator is designed to enhance acoustic signals at a specific frequency by constructing a tube whose length is adjusted to be one-quarter of the wavelength of that frequency. This arrangement allows the device to benefit from constructive interference, significantly boosting the signal’s strength at the resonant frequency. The resonant frequency (*Fr*) for such a resonator is calculated using Eq. (2). Here, “*Fr*” is the resonant frequency, “*c*” represents the speed of sound, and “*L*” is the tube’s length, adjusted to a quarter of the wavelength^47^.

2 $${Fr}=\frac{c}{4L}$$

To understand why this configuration causes resonance, consider that when a sound wave enters the tube, it travels to the end and returns. If the tube length is exactly one-quarter of the wavelength, the reflected wave will be in phase with the incoming wave, causing the waves to reinforce each other. This results in a standing wave pattern with a node (point of minimum displacement) at the closed end of the tube and an antinode (point of maximum displacement) at the open end. This resonance phenomenon in a quarter-wavelength tube enhances the acoustic energy at the resonant frequency, making it particularly effective for energy harvesting. The quarter-wavelength resonator’s ability to amplify specific frequencies means it can be tailored to capture energy from prevalent environmental sounds, thereby improving the efficiency of acoustic energy harvesters (AEHs) like PENGs.

Cantilevers are fundamental structural elements in engineering and physics, characterized by being anchored at one end while free to vibrate at the other. This unique configuration enables cantilevers to convert mechanical energy into various forms through oscillations. When a force is applied to the free end, the cantilever bends, generating tensile and compressive stresses along its length. These stresses result in mechanical deformation, which can be harnessed for various applications. A crucial aspect of cantilever operation is their resonant frequency, determined by the cantilever’s material properties, length, and cross-sectional area. The resonant frequency is pivotal for efficiency because, at resonance, the cantilever vibrates with maximum amplitude. This maximized vibration significantly enhances the stress applied to any materials integrated with the cantilever, optimizing mechanical energy conversion. The resonant frequency (*Fr*) can be calculated using Eq. (3). where *k* is the stiffness of the cantilever and *m* is the effective mass.

3 $${Fr}=\frac{1}{2\pi }\sqrt{\frac{k}{m}}$$

**Fig. 1:**
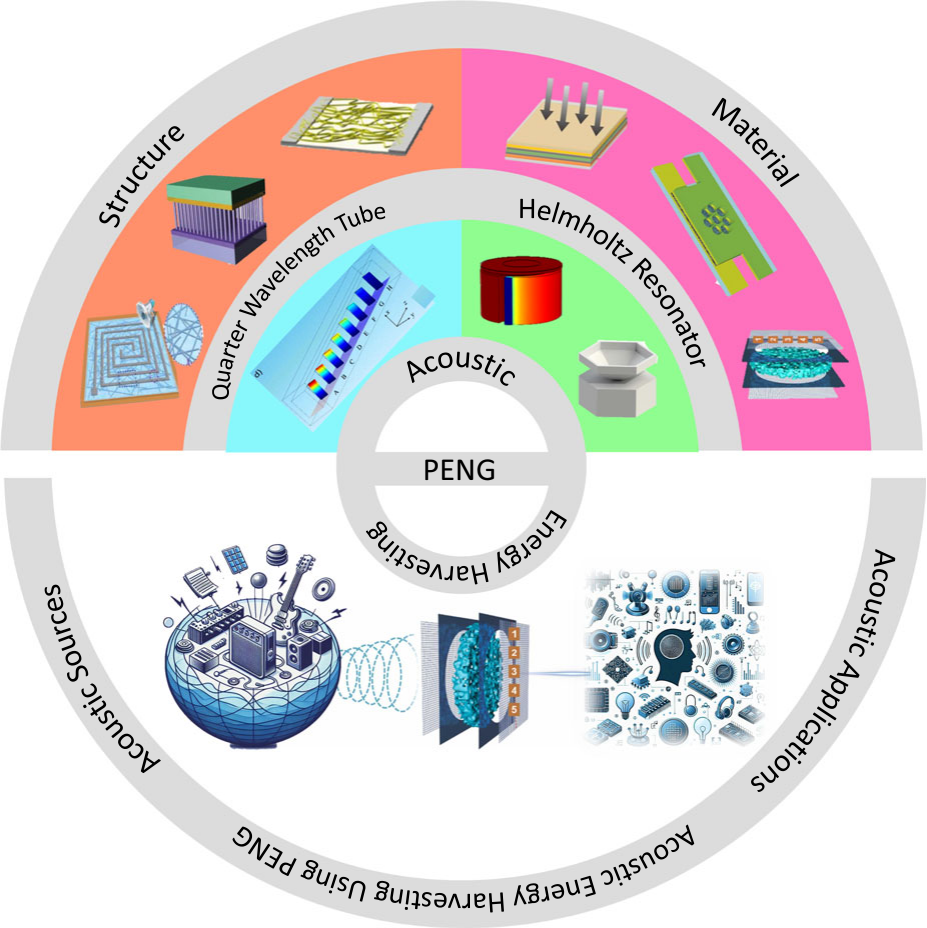
Schematic representation of the key elements in acoustic energy harvesting using Piezoelectric Nanogenerators (PENG). The figure illustrates the focus on material development^60^. Copyright © 2022 Elsevier Ltd. All rights reserved^64^. Copyright © 2020 Elsevier Ltd. All rights reserved^65^. Copyright © 2021 Elsevier B.V. All rights reserved. It also highlights structural innovations^68^ Copyright © 2019, The Royal Society of Chemistry^69^. Copyright © 2014 American Chemical Society^70^. Copyright © 2010 WILEY-VCH Verlag GmbH & Co. KGaA, Weinheim. Also the use of Helmholtz resonators^80^. Copyright © 2018 Author(s), All article content, except where otherwise noted, is licensed under a Creative Commons Attribution (CC BY)^81^. Copyright © 2018 Elsevier Ltd. All rights reserved. and quarter-wavelength tubes^75^ Copyright © 2013 IOP Publishing Ltd, designed to enhance energy capture. The diagram identifies various acoustic sources, ranging from environmental noises to mechanical vibrations, PENGs can harness that. Additionally, the figure outlines the potential applications of PENGs in wearable devices, medical implants, and urban infrastructure, showcasing their versatility in different fields

This study ventures into the evolving field of acoustic energy harvesting, emphasizing the role of PENG. It aims to conduct a detailed examination of various harvesting devices, distinguished by their structural and material characteristics, to evaluate essential performance indicators like voltage, current, and power output. Additionally, this analysis will explore the practical uses of these devices, highlighting their potential impact in real-world scenarios. Moving beyond mere theoretical exploration, the paper will also contemplate the future trajectory of acoustic energy harvesting. This includes an assessment of the current state of the field and speculative insight into potential innovations and unexplored avenues. Figure 1 provides a comprehensive overview of the PENG acoustic energy harvesting process, illustrating the different methodologies and approaches employed in this domain.

## Acoustic PENG

Acoustic PENGs have gained interest in energy harvesting as a viable approach for converting acoustic energy into useable electrical power. PENGs are distinguished by their extraordinary sensitivity, which enables them to detect even the most minor mechanical vibrations, including low-frequency noises that are challenging to harness by the standard energy-harvesting methods. PENGs are distinguished by their nanoscale design, allowing smooth integration into tiny devices without adding substantial bulk or weight. Researchers and scientists actively explore various aspects of acoustic PENG technology to unlock its full potential. Developing and utilizing advanced materials with enhanced piezoelectric properties have been a focal point. Noteworthy examples include materials such as polyvinylidene fluoride (PVDF), PZT plates (lead zirconate titanate), and the growth of ZnO nanowires^48,49^. These materials promise to significantly improve the efficiency of PENGs and expand their capabilities. Quarter-wavelength configurations have emerged as a particularly intriguing area of study. These configurations leverage the resonance phenomenon occurring at a quarter of the wavelength to optimize the capture of acoustic energy. Researchers are diligently exploring different geometries and materials to fine-tune quarter-wavelength PENGs, especially in situations where acoustic energy sources can be controlled or adapted to match the resonance frequency. Integrating HR into PENG designs has proven an effective strategy for enhancing performance. HR provides the advantage of precise frequency tuning, enabling targeted energy harvesting from specific sources^50^. This resonator-based approach has expanded the capabilities of PENGs, making them even more efficient and adaptable in various applications. The research landscape in acoustic PENG technology is continuously evolving, with scientists exploring novel mechanisms and structures inspired by nature. These biomimetic designs promise to improve energy conversion efficiency and expand the potential applications of PENG technology into unexpected domains.

### Different material-based PENG

Piezoelectric materials have a distinctive property whereby they may produce an electric charge when subjected to mechanical stress. This classification of materials encompasses a diverse range of naturally occurring and artificially produced substances^51^. One example of a natural material with piezoelectric capabilities is quartz, which has been used for generations in many applications. Besides quartz, some other natural substances, including Rochelle salt, topaz, cane sugar, and certain forms of bone, have piezoelectric properties^52^. Some ceramics have been identified to possess piezoelectric characteristics in synthetic materials. PZT is well recognized as a prevalent choice among piezoelectric ceramic materials. PZT has a superior piezoelectric constant to quartz, resulting in a greater electrical charge or voltage capacity. This characteristic makes PZT a very desirable material for many industrial applications. PVDF and polyethene terephthalate (PET) have been extensively investigated within the field of polymers. While PET does not possess intrinsic piezoelectric qualities, researchers have successfully devised methods to generate piezoelectric characteristics in this material. This breakthrough has significantly broadened the range of possible applications within piezoelectricity. PVDF is recognized as an extraordinary material in PENGs due to its distinct characteristics. One of the most notable characteristics of this material is its significant piezoelectricity, especially in the beta (β) phase^53^. The high piezoelectric coefficient shown by PVDF in its β-phase enhances energy conversion efficiency, thus leading to improved nanogenerator performance. The flexibility demonstrated by PVDF is a significant characteristic that makes it suitable for various applications. The inherent characteristic of this feature enables the material to be easily transformed into multiple forms and dimensions, offering a notable level of flexibility in design. PVDF’s mechanical durability and strength enhance its reliability as a material. PVDF has remarkable mechanical resilience, rendering it well-suited for many applications, including frequent or elevated degrees of physical contact or motion^54,55^. This section highlights the most commonly used materials for PENGs, focusing on their unique properties and advantages that make them suitable for various applications in piezoelectric energy harvesting.

**Fig. 2:**
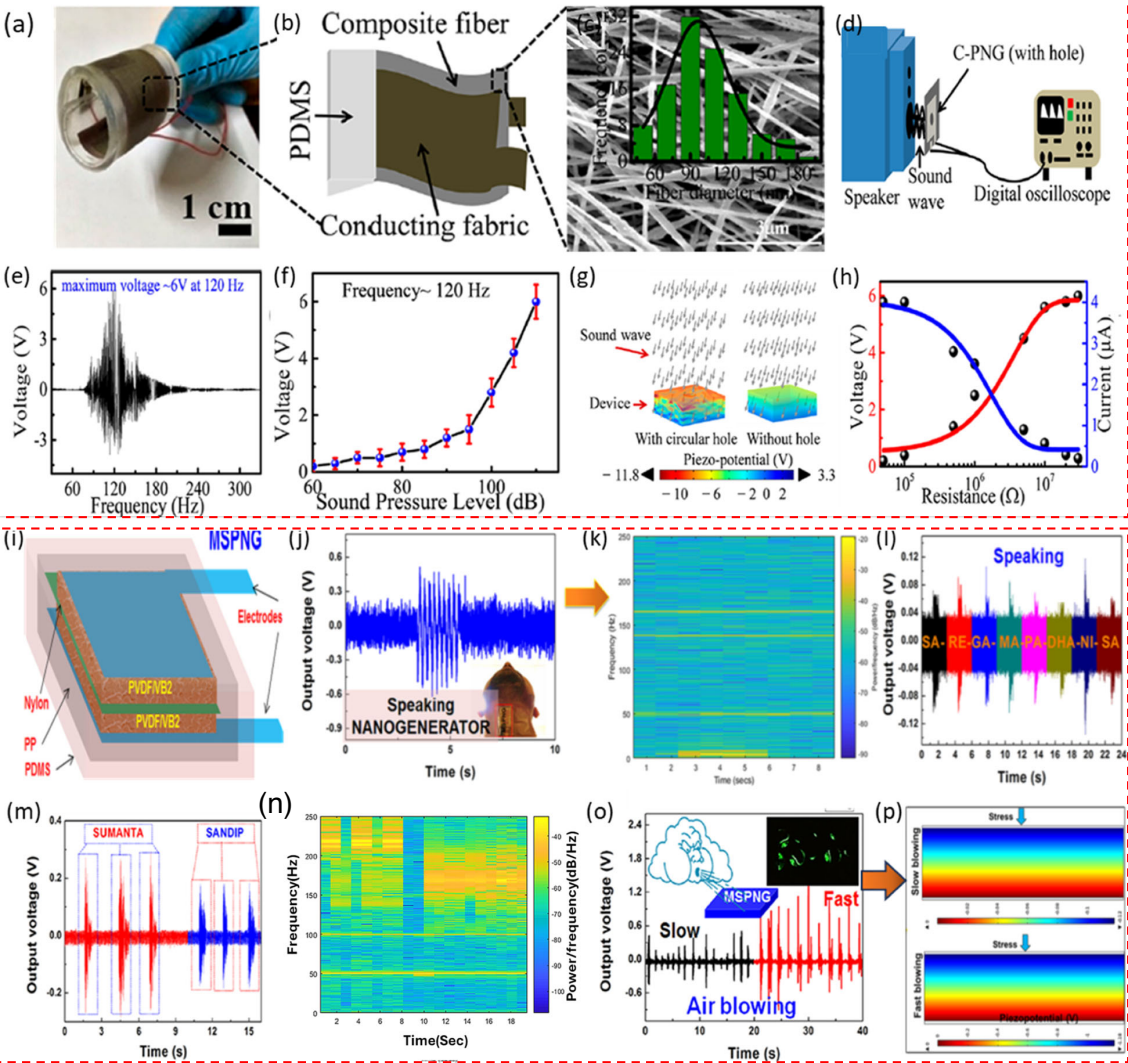
Designs and performance of PVDF-based acoustic piezoelectric nanogenerator. **a** Image of the flexible C-PNG featuring a layered structure that includes a PVDF film, electrodes, and an encapsulating PDMS layer^56^. **b** the fabrication process of C-PNG^56^. **c** FE-SEM image of the composite nanofiber^56^. **d** Schematic representation of the measurement setup used to assess the C-PNG’s response to sound waves, including a speaker and digital oscilloscope^56^. **e** Effect of frequency on the output voltage^56^. **f** Effect of SPL on the output voltage at frequency of 120 Hz^56^. **g** The impact of holes and without on the performance of the device^56^. **h** Changes in output voltage and current with varying resistance values^56^. Copyright © 2021 American Chemical Society. **i** Structure of the MSPNG, consisting of layers including a glass slide, PVDF/VB₂ film, copper tape, transparent nylon woven fabric, polypropylene (PP) tape, and PDMS^57^. **j** Output voltage generated by the MSPNG during vocalization, demonstrating its capability to capture sound-induced vibrations^57^. **k** STFT analysis of the device’s response to vocalization, providing a time-frequency representation of the captured signals^57^. **l** Recorded voltage signals obtained during the articulation of the phrase “SA-RE-GA-MA-PA-DHA-NI-SA”^57^. **m** Voltage output recorded during the vocalization of two words alongside^57^. **n** The associated spectrograms of the vocalization, depicting frequency content over time^57^. **o** Output voltage response of the MSPNG when exposed to different airflow speeds, showing variations in power output between slow and fast air blowing^57^. **p** Visualization of the distribution of potential and displacement across the MSPNG structure, showing areas of high and low piezoelectric response during deformation^57^. Copyright © 2019 Elsevier Ltd. All rights reserved

Roy et al.^56^ introduced a novel approach for fabricating a two-dimensional metal-organic framework (MOF) using cadmium iodide (CdI2) and a 1,5-diaminonaphthalene (NAP) linker through a straightforward and economical mixed solvent layering technique. Following the fabrication of the MOF, it was integrated with PVDF to create electrospun nanofibers, which were then utilized to develop a porous nanogenerator with a composite fiber structure (C-PNG). The creation of [CdI2(NAP)]_n_ and its associated polymer configuration and the FE-SEMG image is detailed in Fig. 2a–c. The functionality of this device was assessed through a battery of mechanical tests, including compression, bending, twisting, and exposure to sound waves within a frequency spectrum of 20–330 Hz. The assembled nanogenerator comprises an operative layer of MOF/PVDF nanofibers placed between two indium tin oxide-coated electrodes, enabling the capture of electrical energy from mechanical vibrations caused by sound or pressure. To enhance the device’s interaction with sound waves, circular openings with a diameter of 1 cm were crafted on the PET films, allowing the nanofibers direct exposure to sound from a nearby speaker, as shown in the schematic (Fig. 2d). This setup proved capable of generating an open-circuit voltage (V_oc_) of up to 6 V under a sound frequency of 120 Hz and a sound pressure level (SPL) of 110 dB. The research team also explored how the device’s piezoelectric output varied with sound frequencies ranging from 20 Hz to 330 Hz at a constant SPL of 110 dB, chosen for its alignment with the threshold of human discomfort from loud sounds. They observed a peak voltage output at 120 Hz, demonstrating a direct relationship between frequency and voltage output that decreased beyond this peak, as shown in Fig. 2e. Additionally, they examined the nanogenerator’s response to different SPLs while keeping the frequency steady at 120 Hz (Fig. 2f). Through finite element method (FEM) simulations, they assessed the performance of the nanogenerator with a central circular aperture in the electrode, noting an increase in voltage output compared to configurations without such apertures (Fig. 2g). The device showcased exceptional precision in detecting a wide range of acoustic vibrations and converted them efficiently into electrical energy, achieving a sensitivity of 0.95 V/Pa. Moreover, an analysis of the device’s instant power output against varying load resistances revealed a maximum power output of 6.25 μW at a 1 MΩ resistance when exposed to a 120 Hz frequency and 110 dB SPL sound waves, with the output voltage increasing to the resistance and the current showing an inverse trend (Fig. 2h). Karan et al.^57^ utilized Vitamin B2 (VB2) as a pivotal enhancer for PVDF, a material renowned for its piezoelectric properties. This innovation primarily hinges on stabilizing PVDF’s β-phase, a critical factor for maximizing piezoelectric effectiveness, through VB2’s interaction with PVDF’s molecular framework, particularly by forming hydrogen bonds. This interaction significantly amplifies the material’s piezoelectric capabilities (Fig. 2i). VB2 stands out for its biodegradability and biocompatibility, making it an ideal component for environmentally sensitive applications or those involving direct contact with biological tissues. These attributes, coupled with VB2’s thermal stability and cost efficiency, pave the way for its application in various technologies. The research proposes developing an organic, multimodal, biocompatible energy harvester, incorporating a robust nylon mesh to enhance its utility and performance. A particularly notable application of this technology is its deployment in a throat-attached nanogenerator that excels in generating electrical power from the vibrations of vocal cords during various activities, such as speaking, swallowing, or gargling. The device’s output voltage variations during these activities were meticulously analyzed using the Short-time Fourier Transform (STFT) to produce a spectrogram that maps the frequency content changes over time, offering invaluable insights into vocal cord dynamics (Fig. 2j, k). This Microscale Scallop-Structured PENG showcases an exceptional capacity for converting spoken words and musical vibrations into electrical signals, displaying a unique response to different sound inputs, including musical instruments and the distinct notes of Indian classical music (Fig. 2l). The AEH demonstrates its capability during speech, capturing energy from various sounds such as speaking different letters, words like “SUMANTA” (≈0.27 V), and “SANDIP” (≈0.21 V), which generate corresponding electrical signals (Fig. 2m). Furthermore, the time-dependent frequency spectrum of the output signals, analyzed using the STFT, reveals the variation in power spectral density with time and frequency for each of the above sounds (Fig. 2n). It also demonstrated a pronounced sensitivity to wind energy, effectively harvesting power from varying speeds of air blown into it, with its prowess showcased by activating six green light-emitting diodes (LEDs) with faster airflows (Fig. 2o). To further understand the device’s electromechanical responses, FEM-based simulations were conducted, which corroborated the experimental findings by illustrating a uniform pressure distribution across the device and matching piezoelectric potentials for different blowing speeds, thus validating the practical observations with theoretical models (Fig. 2p).

**Fig. 3:**
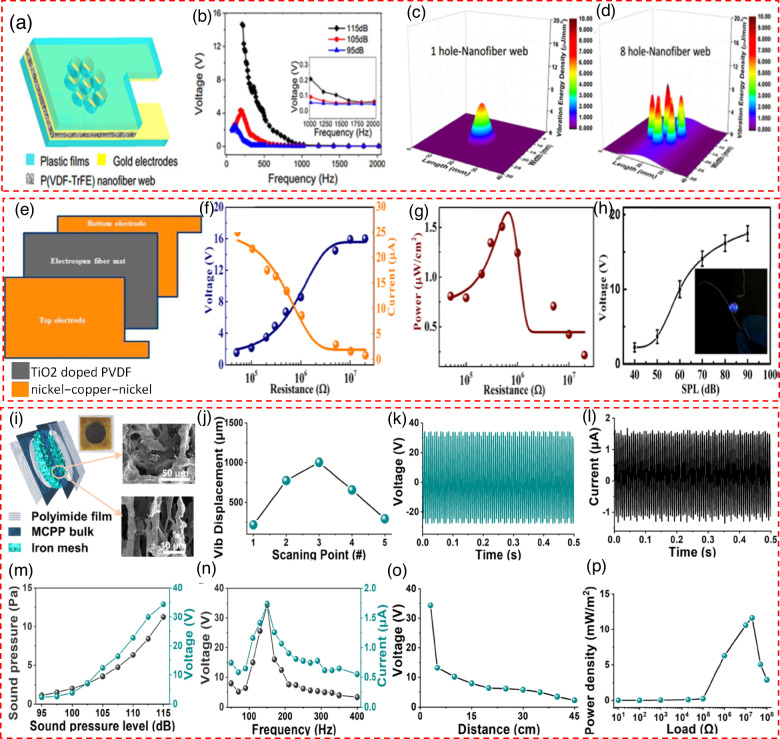
Design and performance of PVDF-based acoustic piezoelectric nanogenerator. **a** Structure of the device, showing a layered configuration with PVDF-TrFE nanofiber webs, gold electrodes, and plastic films^58^. **b** Impact of the frequency on the device’s voltage output (inset enlarged voltage outputs for the frequency range of 1000-2000 Hz^58^). Nanofiber webs’ vibration energy density modeled in (**c**) one-hole^58^ and (**d**) eight-hole devices under an SPL of 115 dB and frequency of 210 Hz^58^. Copyright © 2017 Elsevier Ltd. All rights reserved. **e** Schematic of the nanogenerator, highlighting the structure consisting of a TiO₂-doped PVDF layer sandwiched between two nickel-copper-nickel electrodes^59^. **f** Changes in the output voltage and current of the nanogenerator across different resistance values, demonstrating its performance under varying loads^59^ as well as the (**g**) Power density of the nanogenerator as a function of load resistance, showing the optimal resistance for maximum power output^59^. **h** Output voltage variation with different sound pressure levels (SPL)^59^. Copyright © 2018 American Chemical Society. **i** Structure of the device featuring an MCPP bulk, an iron mesh electrodes, and a polyimide film for protection^60^. **j** The vibration amplitude of the material measured at five evenly spaced points along its diameter using a laser Doppler vibrometer (LDV). The results indicated that point #3, positioned at the center of the material’s surface, showed the highest vibration amplitude, reaching up to 1050 μm, compared to points #1 and #5 at the edges^60^. **k** The output voltage^60^ and (**l**) Current generated by the device over time^60^. **m** The effect of sound pressure on the output voltage and current^60^. **n** Output voltage and current response of the MSPNG across a frequency range of 0–400 Hz, demonstrating variations in performance at different frequencies.^60^. **o** Output voltage variation at different distances from the sound source, demonstrating sensitivity to distance^60^. **p** Changes in output power density of the nanogenerator when subjected to different load resistances, showing the relationship between load and power generation^60^. Copyright © 2022 Elsevier Ltd. All rights reserved

Lang et al.^58^ delved into the acoustoelectric conversion capabilities of nanofiber devices made from electrospun poly(vinylidene fluoride-trifluoro ethylene) (PVDF-TrFE), focusing on their application in energy harvesting. These devices are crafted by embedding a web of PVDF-TrFE nanofibers, primarily featuring the β-crystal phase, between electrodes coated with gold on PET films, thus forming a piezoelectric structure (Fig. 3a). The team conducted experiments to assess how different factors affect the energy conversion efficiency of these devices, discovering that sound frequency notably impacts the voltage output of the nanofiber devices, with significant responses to low-frequency sounds (Fig. 3b). An exploration of the device’s response to frequencies over 1000 Hz is detailed in the inset of (Fig. 3b), revealing an optimal output at 210 Hz when subjected to a SPL of 105 dB, and a broader response range extending up to 1000 Hz at an SPL of 115 dB. To enhance the interaction between sound waves and the nanofibers, the study experimented with introducing holes in the PET film electrodes, allowing direct sound wave penetration. Devices configured with eight through holes demonstrated superior energy outputs to those with a single hole or standard piezoelectric films, as shown in (Fig. 3c, d). The research further explores the impact of the number and size of holes, nanofibers’ diameter, and the nanofiber web’s thickness on performance. It was observed that devices crafted from nanofibers with a uniform diameter of 240 ± 40 nm showcased the highest energy outputs and lowest internal resistance, with a noted decline in output as the fiber diameter increased. FEM simulations conducted with COMSOL Multiphysics 3.5a shed light on the vibrational behaviors of the PET film devices when subjected to acoustic waves, mainly focusing on the effect of the number of holes on acoustoelectric responses. These analyses reveal that, beyond an SPL of 105 dB, the PET film in devices with eight holes vibrated with significantly more energy compared to those with a single hole, indicating that multiple holes facilitate a more efficient sound wave interaction, thereby enhancing the device’s vibration energy and its acoustoelectric conversion potential. Alam et al.^59^ approach was investigated to improve the characteristics and mechanical performance of the piezoelectric β-phase. The process encompassed the integration of titanium dioxide (TiO_2_), a semiconductor characterized by a wide indirect band gap. c is widely recognized for its remarkable thermal and chemical stability, superior light-to-electricity conversion efficiency, and capacity to maintain optimal performance over prolonged durations. The integration of various techniques has demonstrated the potential to enhance the applicability of PVDF for energy harvesting and storage purposes. The current study involved the development of a nanogenerator (NG) that is both flexible and wearable. This was achieved using a PVDF/TiO_2_ electrospun fiber mat positioned between two electrodes (see Fig. 3e). This study aimed to gather and utilize mechanical energy obtained from human activities and acoustic vibrations frequently encountered daily. The graphical representation of the fluctuations in output voltage (V), short-circuit current (I), and power (P) across different load resistances (R) can be observed in Fig. 3f, g. The voltage gradually increases until it reaches its maximum magnitude, whereas the current experiences a gradual decrease. Moreover, it is noteworthy that the power density demonstrates a peak value. Furthermore, when subjected to acoustic vibrations, the nanogenerator (NG) reflects the ability to directly supply power to a blue LED. The NG produces an output voltage when music is played. The voltage magnitude gradually increases as the SPL rises, ultimately reaching a saturation point at a threshold of 90 dB. Figure 3h depicts achieving a notable maximum output peak-to-peak voltage measuring 17.5 V.

Yu et al.^60^ innovatively merged piezoelectric and triboelectric principles to create a device capable of efficiently capturing sound energy across a broad frequency spectrum. This device is characterized by an intricate internal composition that includes a porous structure made of multi-walled carbon nanotubes (MWCNTs) and PVDF-TrFE aerogel paired with a polydimethylsiloxane (PDMS) tympanum. Their publication delineates the procedure for fabricating a distinctive composite aerogel embedded with a PDMS tympanum. This process begins with gradually adding Sodium Carboxymethyl Cellulose (SCMC) into an acidic aqueous solution, followed by mechanical stirring at 60 °C. This step is succeeded by sequentially blending a PVDF-TrFE and sodium dodecyl sulfate emulsion in deionized water and a dispersion of functionalized MWCNTs into the SCMC mixture. The blend is then cooled in an ethanol/liquid nitrogen bath and freeze-dried, setting the stage for the aerogel’s formation. A subsequent thermal treatment at 320 °C for 30 min, followed by rapid cooling in liquid nitrogen, enhances the piezoelectric traits by promoting the β-phase transition in PVDF-TrFE. Dipping the aerogel in varied concentrations of PDMS in xylene under vacuum embeds the PDMS tympanum within the pores, which is then dried at 65 °C to remove xylene and cured at 100 °C, culminating in a porous aerogel bulk with an embedded PDMS tympanum (refer to Fig. 3i). This study introduces an aerogel demonstrating superior piezoelectric properties, positioning it as an effective sound energy harvesting nanogenerator. The creation of a three-dimensional network through SCMC, MWCNTs, and PVDF-TrFE facilitates a continuous porous framework ideal for sound wave diffusion and scattering, thereby inducing pore wall vibrations (Fig. 3i). Vibration amplitude measurements, taken at five equidistant points across the diameter using a laser Doppler vibrometer, revealed the highest vibration at the central point, registering at 1050 μm (Fig. 3j). This aerogel effectively converts acoustic energy into mechanical vibrations, which are then transformed into electrical energy by an acoustic-driven nanogenerator (ANG), leveraging both piezoelectric and triboelectric effects. The ANG showcased optimal electrical output—34.4 V and 1.74 μA—under a 150 Hz sound frequency at 115 dB (Fig. 3k, l). As the SPL shifts from 100 dB to 115 dB, the associated sound pressure increases from 2 Pa to 11.25 Pa. This change causes the MCPP ANG’s output voltage to rise significantly, from 3.2 V to 34.4 V (Fig. 3m). Higher sound pressure results in greater surface strain on the material, which facilitates the conversion of additional energy into resonant mechanical energy. The enhanced resonance amplitude subsequently alters the dipole moment considerably, revealing a roughly linear relationship between the sound pressure and the output voltage. Also the ANG’s performance, demonstrating significant responsiveness across a frequency range of 110–400 Hz, exemplifies its capacity to react to various sound frequencies (Fig. 3n). Additionally, the generator’s output voltage displayed a decrease as the distance from the sound source increased, with a peak power density at the optimum distance of 11.62 mW/m^2^ when connected to a 20 MΩ load (Fig. 3o, p), illustrating its efficiency in sound energy harvesting across diverse conditions.

Xu et al.^61^ used embossing templating to create patterns on electrospun PVDF nanofibers, developing a three-dimensional structure with a wave-like morphology. The device demonstrated significant piezoelectric responses in both the longitudinal and transverse directions. PVDF nanofibers were produced using electrospinning technology. The voltage response (*V*_oc_) to auditory stimulation at a frequency of 100 Hz and an SPL of 110 dB displayed fluctuations within a range of ±2.8 mV, closely mirroring the acoustic stimulation. The *V*_oc_ (open-circuit voltage) response of the piezoelectric nanofiber device was shown to be impacted by both the acoustic frequency and SPL. A sequence of V_oc_ responses to auditory stimulation was found, where the SPL remained constant at 110 dB, but the frequencies ranged from 40 to 280 Hz. The study used *V*_oc_ responses obtained from a flat-type device for comparative analysis. The findings demonstrated that, in the case of a particular sound frequency, the *V*_oc___pp_ response exhibited superior performance in the wave-shaped device compared to the flat-type device. This observation confirms the enhanced sensitivity of the 3D structured sensor. Both devices showed a significant decline in *V*_oc___pp_ response beyond 200 Hz. It is essential to note that both acoustic sensors exhibited high responsiveness to sound waves with low and medium frequencies. This observation suggests that these sensors have promise for use in various industrial and public transportation environments, where the prevailing sound frequencies often fall within the range of 40–200 Hz. Mahanty et al.^62^ have notably increased the electroactive phase content in a PVDF nanofiber produced by electrospinning due to the combined influence of interfacial interactions between Mg salts and the PVDF chain and the use of mechanical stretching during fiber collection utilizing a high-speed rotating collector. In addition, a novel all-fiber nanogenerator has been developed using nanofibers based on PVDF-Mg salt. This nanogenerator exhibits excellent sensitivity and can detect noises at low frequencies. Under SPLs of 80 dB and 120 dB, and a frequency of 130 Hz, AAPNG produced periodic open-circuit output voltages with peak values of 2 V and 3 V, respectively. The enhanced energy scavenging capabilities of AAPNG may be ascribed to its higher piezoelectric charge coefficient (*d*_33_ = 33.6 pC N^−1^) in comparison to pristine PVDF (*d*_33_ = 22 pC N^−1^). In this context, the symbol d_33_ denotes the piezoelectric voltage coefficient. The d33 value of the PVDF-Mg nanofibers has a much greater magnitude than a range of other nanofibers based on PVDF. Sun et al.^63^ developed a piezoelectric acoustoelectric nanogenerator by constructing a combined fiber membrane of PVDF-ZnO with a hierarchical microstructure. The manufacturing procedure included the use of electrospinning and hydrothermal processes. The PVDF-ZnOANG exhibited its maximum output voltage of 1.7 V when exposed to a sound frequency of 140 Hz at an SPL of 116 dB. The signals that were measured have been verified to be piezoelectric outputs that are produced by the PVDF-ZnOANG. The PVDF-ZnOANG demonstrated a much higher output voltage, almost three times greater than that of PVDFANG, when subjected to identical circumstances. This notable disparity in performance may be ascribed to the hierarchical structure of the PVDF-ZnOCFM and the synergistic effects arising from the combined semiconductor and piezoelectric capabilities of the ZnO nanorods. The effects of external resistance load on the voltage and current outputs of the PVDF-ZnOANG were also investigated by the researchers. The researchers noted that there was a positive correlation between voltage outputs and external resistance, whereas a negative correlation was discovered between current outputs and external resistance. The PVDF-ZnOANG material demonstrated its peak power output when an external resistance of 700 kΩ was applied. This configuration yielded an output voltage of 1.12 V and an output current of 1.6 µA, resulting in a maximum power output of 1.792 µW.

**Fig. 4:**
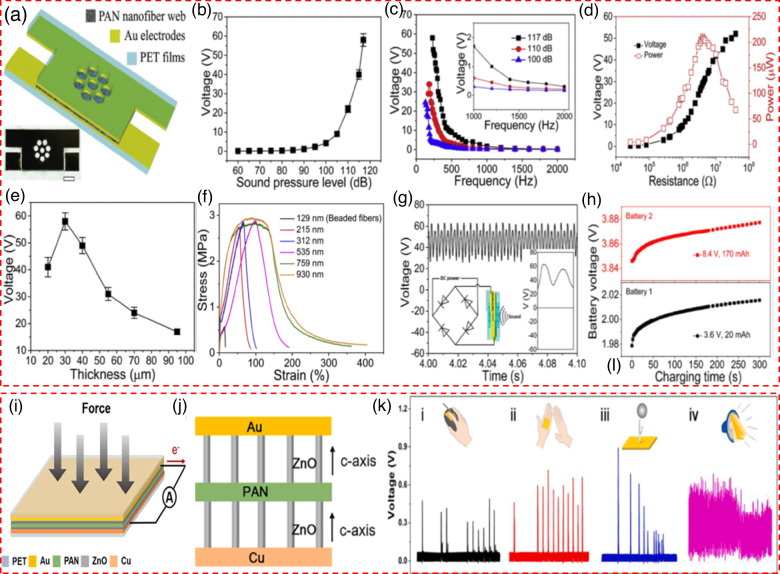
Exploring material based acoustic piezoelectric nanogenerator: design and performance evaluation. **a** Illustration of the PENG structure, highlighting the PAN nanofiber web, Au electrodes, and PET films^64^. Effects of (**b**) Relationship between the sound pressure level (SPL) and output voltage, showing a significant voltage increase at higher SPLs^64^. **c** Output voltage response across different frequencies, indicating how the PENG’s output varies with changes in the frequency of the applied sound^64^. **d** The influence of external resistance on the nanogenerator’s power output and voltage, showing the optimal resistance for maximum power generation^64^. **e** Impact of varying nanofiber thickness on the output voltage, revealing a peak performance at 30 µm^64^. **f** Stress-strain behavior for different PAN fiber diameters, showing how fiber diameter affects mechanical properties^64^. **g** Rectified voltage output; Inset: Diagram of the circuit used to convert AC signals into DC^64^. **h** Charging curves of two different batteries using the nanogenerator, illustrating the charging efficiency and capacity for each battery^64^. Copyright © 2020 Elsevier Ltd. All rights reserved. **i** Detailed structure of the ZnO nanofiber-based nanogenerator, including layers of PET, Au, PAN, ZnO, and Cu, designed for efficient energy conversion^65^. **j** Side view of the PENG structure, showing the arrangement of ZnO nanowires between the PAN layer and the electrodes^65^. **k** Voltage response of the PENG under various practical applications^65^. Copyright © 2021 Elsevier B.V. All rights reserved

Shao et al.^64^ developed an innovative method for converting noise into electrical energy by employing electrospun polyacrylonitrile (PAN) nanofiber membranes, first in utilizing PAN for such applications. Their findings demonstrate that PAN nanofibers outperform traditional PVDF in acoustoelectric conversion efficiency. As detailed in Fig. 4a, the device comprises a PAN nanofiber membrane layer sandwiched between two gold-coated PET films. The study explores how SPL affect the device’s voltage output at a fixed sound frequency of 230 Hz (Fig. 4b). It was observed that voltage output increased from 0.12 V at 60 dB to 1.15 V at 90 dB and surged to 58 V as SPL rose to 117 dB. Figure 4c further investigates the impact of sound frequency on voltage output at a constant SPL of 100, 110, and 117 dB, revealing a decline in output with increasing frequency. Despite limitations in testing lower frequencies due to speaker constraints, the device showed potential for electricity generation at reduced SPLs and frequencies. Moreover, under conditions of 117 dB SPL and 230 Hz frequency, the device’s voltage output initially increased and then decreased with a higher load resistance, ranging from 27 kΩ to 100 MΩ, peaking at an instantaneous power of 210.3 μW, or a power density of 17.53 μW/cm^2^, at a 4 MΩ load (Fig. 4d). The thickness of the PAN membrane was critical for performance, with a thickness increase from 20 μm to 30 μm enhancing voltage from 41 V to 58 V, a notable improvement highlighted in Fig. 4e. However, further thickness increases to 95 μm saw a reduction in performance. Tensile testing of the nanofiber membranes, shown in Fig. 4f, indicated that fiber morphology and diameter significantly affect mechanical properties. Nanofibers with diameters below 535 nm displayed a sharp stress decrease after reaching peak values, with beaded fibers showing lower tensile strength and elongation than uniform fibers. To harness the electrical energy produced by the PAN nanofiber device, a rectifier was employed to transform the AC into DC. The resulting rectified voltage are displayed in Fig. 4g, with the rectifier circuit diagram illustrated in the inset of the same figure. The practical application of this technology in energy storage is demonstrated in Fig. 4h, where capacitors charged by the nanogenerator show voltage increases, the device’s efficacy in charging lithium-ion batteries of 20 mAh and 170 mAh capacities under noise exposure, witnessing voltage boosts within 300 s. This underscores the PAN nanofiber device’s rapid and efficient energy harvesting and storage capabilities, marking a significant step forward in renewable energy technology. Sun et al.^65^ crafted an innovative Schottky DC nanogenerator characterized by its unique layered structure, integrating ZnO nanorod arrays affixed to a PAN film, which is then sandwiched between gold (Au) and copper (Cu) electrodes (Au/ZnO-PAN/ZnO-Cu) as depicted in Fig. 4i. A notable aspect of their design is the uniform crystallographic orientation of the ZnO nanorod arrays (Fig. 4j). The device’s notable DC output power is the result of synergizing the piezoelectric properties of both the PAN film and ZnO nanorods. The Au-ZnO Schottky junction plays a pivotal role, ensuring a one-way electron flow towards the metal electrode, thus boosting the nanogenerator’s output. Additionally, the ZnO nanorods aligned with the Cu electrode serve as a barrier to electron backflow, further amplifying the efficiency of the energy conversion process. The primary objective of their research was to quantify the Au/ZnO-PAN/ZnO-Cu PENG’s efficacy in transforming various mechanical actions into electric power, as detailed in the findings presented in Fig. 4k. Impressively, the nanogenerator registered output metrics—voltage around 0.4 V and current close to 8 μA—when activated by the mechanical force of a mouse click. Beyond quantifying output, the device showcased an ability to differentiate between distinct mouse-click patterns, including single, double, and sustained clicks, demonstrating its nuanced response to varied mechanical stimuli. Furthermore, the conversion of mechanical energy from the act of clapping hands into electrical energy was successfully achieved by the nanogenerator. This led to the generation of an electrical signal with a slightly higher magnitude of ~0.6 V and 10 μA, which can be attributed to the variation in the applied mechanical force. In addition, the nanogenerator exhibited effective electrical signal generation when exposed to the impact of a small rubber ball in a state of unrestricted downward motion. Additionally, it demonstrated a positive electrical reaction to high-frequency oscillations, specifically sound oscillations at a frequency of 200 Hz. The findings of this study indicate that the Au/ZnO-PAN/ZnO-Cu PENG displays a broad spectrum of frequency response when subjected to different types of mechanical stimulation.

Li et al.^66^ provided a report on the production and characterization of a poling-free nanogenerator composed of bio-based PLLA/VB2 materials. A significant enhancement in the crystallinity and molecular orientation of PLLA was reported as a result of the incorporation of VB2 into the polymer mix. As a result, the researchers were able to effectively fabricate a (PLLA)/(VB2) nanogenerator that exhibited enhanced piezoelectric characteristics. The inclusion of VB2 in the PLLA/VB2 composite film within the weight percentage range of 0–15% leads to a significant enhancement in the peak voltage of the nanogenerator. The greatest voltage achieved is 4.51 V, representing a five-fold increase compared to the pure PLLA-based nanogenerator, which generates just 0.88 V. The observed rise in voltage may be ascribed to the merger of VB2 with PLLA, which facilitates the alignment of the PLLA molecular chains and augments the crystalline structure of PLLA. Consequently, this leads to an enhancement in the piezoelectric properties of PLLA. Nevertheless, it is observed that the peak voltage shown by the PLLA/VB2 nanogenerator containing 20 wt% VB2 is comparatively lower in comparison to the PLLA/VB2 nanogenerator incorporating 15 wt% VB2. The observed phenomenon may be attributed to an excessive quantity of VB2, resulting in an uneven distribution within the PLLA material. The determination of the ideal amount of VB2 in the PLLA/VB2 film for achieving optimal nanogenerator performance indicates that a weight percentage of 15% is recommended. In order to optimize the output power of the PLLA/VB2 nanogenerator, an assessment was conducted by systematically altering the load resistance within the range of 0–1000 MΩ. The results indicate that the PLLA/VB2 nanogenerator achieved a maximum peak power density of 75.68 mW cm^−3^ when subjected to a load resistance of 28 MΩ. In contrast, the pure PLLA nanogenerator only attained a power density of 2.64 mW cm^−3^ under a load resistance of 68 MΩ. Hence, it can be shown that the peak power density of the PLLA/VB2 nanogenerator exhibited a substantial increase of around 28.7 times when compared to the pure PLLA nanogenerator.

In the domain of piezoelectric energy harvesting, the selection and innovation of materials are pivotal, reflecting a rapidly evolving landscape where the fusion of material science and engineering plays a crucial role. This section delves into the diverse materials employed in PENG, spanning from natural piezoelectric like quartz and various synthetic polymers such as PVDF, to cutting-edge composite materials. The integration of advanced materials, including MOF and nano-enhanced polymers, significantly amplifies the electrical output and efficiency of PENGs, facilitating their application across a wide array of fields, from wearable electronics to acoustic sensing and energy harvesting. The exploration of nanostructured materials, alongside innovative fabrication techniques for electrodes and energy harvesting membranes, marks significant strides in tailoring PENGs for specific operational frequencies and mechanical stress conditions.

### Structural-based PENG

The fundamental construction of a PENG has two primary components: a piezoelectric material and metallic electrodes. The main part of the device, known as the piezoelectric material, plays a crucial role in generating electrical charges via the use of mechanical stress or deformation^67^. The use of a thin sheet of piezoelectric material in this application is common due to its high piezoelectric coefficient. To facilitate the collection and transportation of electric charges produced, metallic electrodes are positioned on both the upper and lower surfaces of the piezoelectric layer. PENG often have enclosures to protect the sensitive components and improve their operating effectiveness. To offer structural strength and safeguard the inside components from different external factors, such as dust, moisture, and physical injury, the enclosure is often built from a tough and lightweight material. In the following papers, the structural designs presented are unique compared to conventional approaches. These studies explore innovative configurations that diverge from standard practices, offering new insights and potential advancements in piezoelectric energy harvesting.

**Fig. 5:**
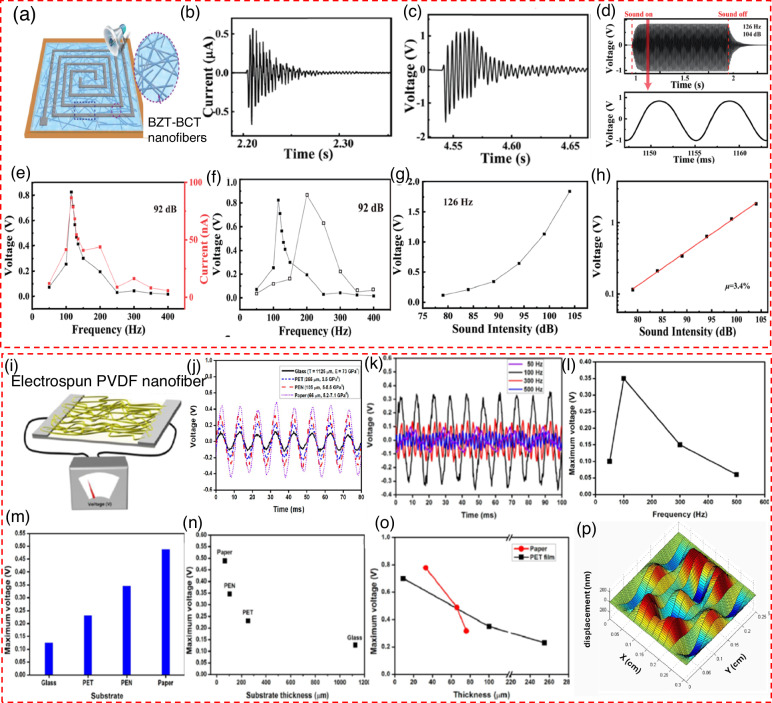
Innovative structures for efficient energy harvesting in acoustic piezoelectric nanogenerator. **a** Structure of the nanogenerator, consisting of BZT-BCT nanofibers arranged in a spiral pattern on a flexible substrate^68^. Example of a single (**b**) output current pulse and (**c**) one output voltage pulse generated by the nanogenerator, demonstrating the device’s response to mechanical excitation, along with the gradual decay of the signal over time as the energy dissipates^68^. **d** Top: Output voltage of the PENG under sound conditions of 126 Hz frequency and 104 dB sound intensity, showing the voltage variation over time bottom: a close-up to see a single pulse of the output voltage^68^. **e** Voltage and current output of the nanogenerator driven by a speaker across various frequencies under 92 dB, indicating how performance changes with frequency^68^. **f** Comparison of the voltage output generated by two different devices, each with a distinct membrane tension, highlighting the influence of tension on performance^68^. **g** Voltage output of the PENG at different sound pressure levels (SPL) when exposed to a fixed frequency of 126 Hz, showing sensitivity to sound intensity^68^. **h** The semi-logarithmic correlation between the dB level and the generated voltage^68^. Copyright © 2019, The Royal Society of Chemistry. **i** Structure of the nanogenerator, featuring an electrospun PVDF nanofiber mat layered on a conductive substrate^69^. **j** Output voltage generated using different substrate materials, such as glass, PET, PEN, and paper, showing their influence on device performance^69^. **k** Patterns of output voltage cycles for different frequencies^69^ and (**l**) Generated voltage displayed against frequency^69^. **m** V_OC_ output comparison for different substrate materials, including glass, PET, PEN, and paper^69^. **n** Relationship between the thickness of each substrate material used and the corresponding output voltage, showing how variations in material thickness impact the generated voltage^69^. **o** Output voltage for paper and PET film materials with different thickness^69^. **p** Mechanical distortion patterns on the surface of the PVDF nanofiber mat caused by acoustic waves, visualized through displacement mapping^69^. Copyright © 2014 American Chemical Society

Cui et al.^68^ introduce a PENG that harnesses BZT-BCT nanofibers for converting noise into electric energy. By embedding BZT-BCT nanofibers within a PDMS matrix, they fabricate a monolayer membrane structure, as shown in Fig. 5a. This design is unique for its ability to vibrate vertically when exposed to sound, with these vibrations causing the BZT-BCT nanofibers to cyclically stretch and relax between two electrodes. This action facilitates a steady flow of electrons in the external circuit, responding dynamically to the variations in piezoelectric potential. The PDMS not only fills the voids in the BZT-BCT fabric but also reinforces the structure, enhancing its durability while preserving its lightweight and thin profile. The electrical output of the PENG was evaluated using a manual drumming test, where the force of each drum hit slightly varied, influencing the voltage peaks as recorded. The outcomes, illustrated in Fig. 5b, c, present the output current and voltage as damped waves, mirroring the drumhead’s damped vibrations with an electrical signal frequency closely aligned with the drum’s natural frequency. Subjecting the PENG to a 126 Hz and 104 dB sound for 1 s showed a peak output voltage of 1.0 V, with the system stabilizing in just 0.08 s (Fig. 5d). Further testing across a frequency range from 50 Hz to 400 Hz, maintaining a sound level of 92 dB, revealed how the output signal’s amplitude varied with the frequency of the acoustic driving wave, demonstrating a clear link between the output signal and the frequency, as seen in Fig. 5e. This relationship allowed for the detection of acoustic waves, pinpointing a resonance frequency at 115 Hz, where the device’s output voltage and current peaked at 0.45 V and 44 nA, respectively. On the other hand, the lowest recorded outputs at 400 Hz were 9 mV and 3 nA. Adjusting the tension in the BZT-BCT membrane increased the resonant frequency to 200 Hz, indicating the membrane’s tension directly affects its frequency response (Fig. 5f). The relationship between sound intensity and output voltage is a key factor in understanding the performance of the device. Figure 5g clearly demonstrates this connection, showing how variations in intensity affect the voltage output. To gain deeper insights into this trend, Fig. 5h presents the same data on a semi-logarithmic scale. This representation reveals an almost linear increase. Lee et al.^69^ investigates the impact of various substrates on the piezoelectric properties of electrospun PVDF nano fibers. The primary objective of this study is to enhance the efficiency of energy harvesting from sound waves. This study examines different substrates, which are glass, PET film, polyethylene naphthalate (PEN) film, and paper, and evaluates their influence on the output voltage of energy harvesters based on PVDF. Figure 5i illustrates a schematic representation of the energy harvester based on PVDF nanofibers. The PVDF nanofibers were fabricated using the electrospinning technique, with two aluminum (Al) foil electrodes placed on the substrate. The application of external sound waves resulted in the vibration of the substrate, thereby generating voltage output. the study explored the impact of different substrate materials on the device’s output voltage. Figure 5j compares the voltage output when using glass, PET, PEN, and paper as substrates. The results indicate that each material influenced the generated voltage differently, with paper showing a more favorable response. In Fig. 5k, the voltage cycling profiles are shown for various sound frequencies. These profiles were obtained using a 100 μm thick PET-film substrate, with a sound power level of 100 dB. The output voltage profile remained consistently stable when the lowest frequency (50 Hz) was employed, despite the non-sinusoidal waveform resulting from its small amplitude. The amplitude of the output voltage was less than 100 mV. Conversely, when considering higher frequencies such as 300 Hz and 500 Hz, it was observed that the maximum voltages exhibited fluctuations and their amplitudes consistently remained below 200 mV. The observed variability in voltage output profiles at higher frequencies may be ascribed to a discrepancy between the applied mechanical stress and the relaxation characteristics of the PVDF nanofibers. In contrast, when the input frequency of the sound wave was adjusted to 100 Hz, the system exhibited a remarkably stable oscillation and a notable amplitude of ~350 mV (Fig. 5l). As a result, the researchers made the decision to maintain the input sound wave frequency at 100 Hz. The authors noted a gradual increase in the maximum output voltages (Fig. 5m) as they transitioned from glass to paper-based harvesters. This observation implies that there is a positive correlation between the degree of deformation experienced by a substrate and the resulting output voltage. Furthermore, a distinct and consistent non-linear correlation between the thickness of the substrate and the output voltage was observed (see Fig. 5n). Substrates with reduced thickness, irrespective of their inherent stiffness, exhibited the ability to generate elevated voltages. Consequently, the researchers reached the determination that paper, due to its abundant availability and favorable piezoelectric properties, is the optimal substrate to produce nanogenerators with high efficiency. The scientists produced paper-based energy harvesters of different thicknesses, specifically 76 μm, 66 μm, and 33 μm. The objective of this study was to evaluate the impact of paper thickness on the piezoelectric properties of these devices. It was observed that a decrease in substrate thickness led to a substantial increase in the maximum voltage output, as depicted in Fig. 5o. To clarify, it can be stated that the utilization of thinner paper substrates resulted in an increase in voltage generation within the energy harvesters. The vibration model was employed by the researchers to perform a numerical simulation of the paper substrates. The simulation facilitated their comprehension of the deformation exhibited by the energy harvesters in reaction to the sound waves. Among the various thicknesses considered, it was observed that the paper substrate with a thickness of 33 μm exhibited the most significant deformation when subjected to sound waves (see Fig. 5p).

**Fig. 6:**
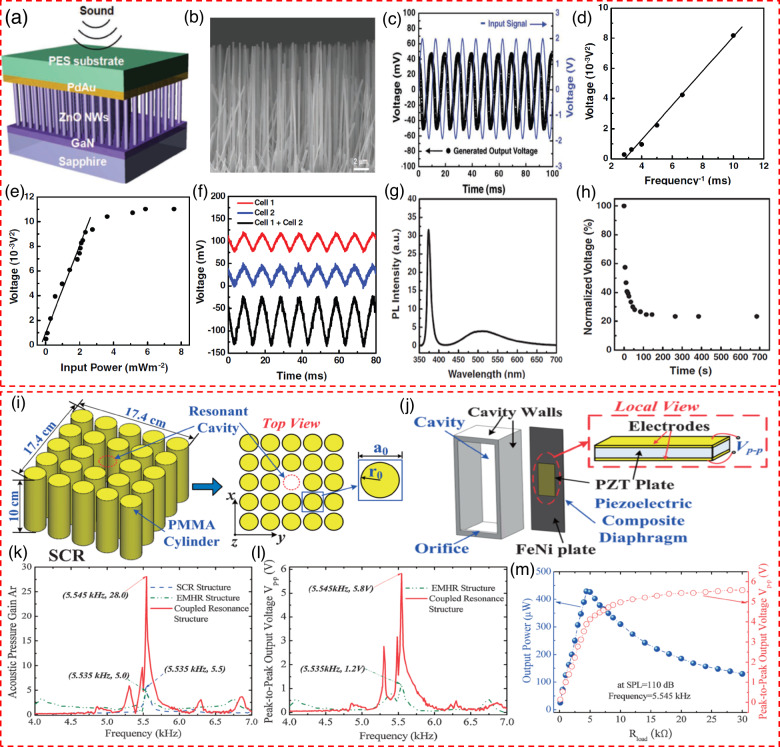
Innovative structures for efficient energy harvesting in acoustic piezoelectric nanogenerator. **a** Structure of the ZnO nanowires-based nanogenerator, consisting of vertically aligned ZnO NWs grown on a GaN substrate, with a Pd/Au layer and a flexible PES substrate for enhanced energy conversion efficiency^70^. **b** FE-SEM image of ZnO NWs, showing the vertically aligned nanowire array and their uniformity^70^. **c** The figure displays plots of the input signal used to generate the sound wave and the resulting output voltage produced by the integrated nanogenerator^70^. **d** Generated voltage as a function of varying acoustic frequency, showing the relationship between frequency and voltage output^70^. **e** Generated voltage vs the input acoustic wave’s input power at 100 Hz^70^. **f** Superposition of output voltages from two cells, showing the combined voltage response when multiple cells are used^70^. **g** Photoluminescence (PL) intensity spectrum of ZnO NWs, indicating the optical properties of the nanowires^70^. **h** Normalized voltage decay of the ZnO nanowire-based nanogenerator over time when exposed to continuous acoustic waves. The plot shows a rapid decrease in voltage output within the initial period, stabilizing to a lower level as time progresses.^70^. Copyright © 2010 WILEY-VCH Verlag GmbH & Co. KGaA, Weinheim. **i** Diagram of the SCR structure, comprising an array of PMMA cylindrical resonators arranged to focus sound waves into a confined region for enhanced energy harvesting^71^. **j** Diagram displaying the EMHR structure, featuring a cavity with a PZT layer sandwiched between electrodes^71^. **k** Acoustic pressure gain as a function of frequency^71^. **l** V_pp_ against frequency^71^. **m** Output power and peak-to-peak voltage of the nanogenerator measured against different load resistances^71^. Copyright © 2013 The Japan Society of Applied Physics

Cha et al.^70^ delve into a nanogenerator built with piezoelectric ZnO nanowires, showcased in Fig. 6a, while Fig. 6b features a field emission scanning electron microscopy (FE-SEM) image of these nanowires. The nanogenerator employs vertically aligned ZnO nanowires as the piezoelectric layer. ZnO nanowire growth is facilitated by thermal chemical vapor deposition on an n-type GaN film, which acts as the bottom electrode on a sapphire substrate. The top electrode and vibration plate, made of a PES substrate coated with PdAu, cover the ZnO nanowire arrays. The ZnO nanowires, with typical dimensions of about 10 μm in length and 150 nm in diameter, are crucial for the device’s operation. Figure 6c depicts the input signal generating the sound wave and the resulting output voltage from the nanogenerator. An input sound intensity of roughly 100 dB (10^−2^ W/m^2^ at 100 Hz) achieves an output voltage amplitude of around 50 mV. Keeping the input sound intensity constant at 100 dB, it was noted that the voltage shares a linear relationship with the sound wave frequency (see Fig. 6d). When the frequency was fixed at 100 Hz, Voltage demonstrated a direct correlation with the input power to the nanogenerator, as illustrated in Fig. 6e. Within the 0–3 mW/m^2^ intensity range, V2 exhibited linear growth, suggesting a power output range from 0 to 0.3 μW for a 1 cm^2^ device area. However, V2 eventually reached saturation beyond this intensity range. A linear superposition experiment with two cells, labeled Cell 1 and Cell 2, generating output voltages of 24 mV and 26 mV respectively, showed increased output when connected in series, totaling 52 mV—essentially the sum of the outputs from the individual cells, as depicted in Fig. 6f. This result confirmed that the signal was indeed generated by the PENG in response to sound, ruling out any artifacts from the measurement apparatus. Figure 6g presents the photoluminescence (PL) of the ZnO nanowires integrated into the nanogenerator. It was found that carriers in the ZnO nanowires significantly contribute to enhancing the piezoelectric potential screening rate. ZnO nanowires, with minimal point defects and grown on GaN, show a negligible lattice mismatch with ZnO, making them highly effective for generating piezoelectric potential under mechanical stimuli like sound waves. Typically, the surfaces of ZnO nanowires are covered by oxygen molecules, creating a depletion region that confines free electrons, thus lowering both carrier density and conductivity. However, UV radiation exposure leads to the dissociation of oxygen molecules, freeing trapped electrons, and thereby increasing the conductivity and carrier density within the ZnO nanowires. Despite these enhancements, the high conductivity of the nanowires might impede the generation of piezoelectric potential. Exposing ZnO nanowires to UV light irradiation, as shown in Fig. 6h, might reduce the output voltage of PENGs activated by sound waves. Yang et al.^71^ introduced an AEH that significantly enhances energy collection from sound waves by employing a coupled resonance structure, integrating an electromechanical HR with a compliant piezoelectric composite plate positioned at the core of a sonic crystal resonator (SCR), as depicted in Fig. 6i, j. The optimal performance of this system is achieved when the re-resonator and the SCR share the same resonant frequencies, enabling the highest level of acoustic resonance coupling. This coupling leads to a substantial increase in acoustic pressure within the system compared to what is observed with individual resonator configurations. The enhanced vibrational activity of the piezoelectric composite, driven by the stronger resonant acoustic waves due to coupled resonance, results in increased output voltage and more efficient energy harvesting. Figure 6k shows how the coupled resonance structure yields multiple peaks in acoustic pressure gain (Ar) across different resonance frequencies, a consequence of the synergistic interactions between the acoustic resonance modes of both the sonic array and the HR. The Ar peaks, particularly the one at 28 at 5.545 kHz, are notably higher—5.1 and 5.6 times greater—than those observed in the separate SCR structure and the electromechanical Helmholtz resonator (EMHR) structure, respectively. Correspondingly, Fig. 6l demonstrates that the output voltage of the harvester exhibits several peaks across the frequency spectrum, mirroring the acoustic pressure gain profile. This characteristic enables the harvester to operate effectively over a wider range of frequencies. At its operational best, with a resonant frequency of 5.545 kHz and an input SPL of 110 dB, while under a load resistance of 1 MΩ, the device achieves a peak-to-peak output voltage of 5.8 V. Further analysis on the impact of load resistance on the device’s performance at the resonant frequency of 5.545 kHz is presented in Fig. 6m. Here, the optimal power output of 429 µW is recorded with a load resistance of 4.4 kΩ, accompanied by an SPL of 110 dB. At this optimal point, the peak-to-peak voltage reaches 3.89 V, showcasing the effectiveness of the coupled resonance structure in maximizing the energy harvesting from acoustic sources.

The exploration of structural designs in PENGs is a cornerstone in the quest for efficient acoustic energy harvesting, emphasizing the meticulous integration of piezoelectric materials with conductive electrodes. This section encapsulates the essence of PENGs, highlighting their reliance on the strategic layering of piezoelectric substrates and electrodes to optimize the conversion of mechanical stimuli into electrical energy. The inclusion of novel materials, such as BZT-BCT nanofibers embedded within PDMS matrices, underscores the innovation within this field, marrying material flexibility with structural integrity to enhance electrical output and device durability. The presented studies delve into various facets of PENG design, from the impact of substrate characteristics on piezoelectric efficiency to the utilization of ZnO nanowires for amplified electrical generation in response to sound waves. These investigations not only broaden the understanding of PENGs’ operational dynamics but also pave the way for the development of more responsive, efficient, and versatile energy harvesters.

### Mechanical modulation

Mechanical dynamics modulation is a crucial method for enhancing energy harvesting efficiency, particularly in applications like acoustic energy harvesting. By manipulating the mechanical properties and resonant behaviors of energy harvesting devices, we can significantly boost their performance. One of the key strategies in mechanical dynamics modulation is the use of resonant structures to amplify mechanical vibrations. Devices like HRs, quarter-wavelength tubes, and cantilever beams are designed to resonate at specific frequencies, thereby maximizing the conversion of mechanical energy into electrical energy. For instance, HRs enhance the acoustic pressure within a cavity, leading to increased vibrations that piezoelectric materials can efficiently capture. Another aspect of mechanical dynamics modulation involves the strategic placement and design of piezoelectric elements within resonant structures. By optimizing the geometry and material properties of these elements, we can achieve higher sensitivity to mechanical inputs and better energy conversion rates. Moreover, the integration of mechanical dynamics modulation with advanced signal processing techniques further enhances the overall efficiency of energy harvesting systems. By analyzing and optimizing the mechanical response of energy harvesters to various environmental inputs, we can develop systems that are not only more efficient but also more adaptable to different energy harvesting scenarios^72^.

#### Cantilever-based PENG

A cantilever-based PENG is a distinct arrangement within the PENG technology framework, which leverages the piezoelectric effect inside a cantilever structure to transform mechanical energy into electrical energy. The cantilever is a structural element resembling a beam, characterized by being fixed at one end and allowing for unrestricted vibration or deflection at the other end. The typical configuration of a cantilever-based piezoelectric energy harvesting device involves the incorporation of a layer of piezoelectric material onto the surface of the cantilever. When subjected to mechanical vibrations or external forces, the free end of the cantilever experiences bending or deflection, resulting in the piezoelectric material connected to it experiencing mechanical stress or deformation. The material exhibits the generation of electrical charges on its surfaces as a consequence of the piezoelectric action.

**Fig. 7:**
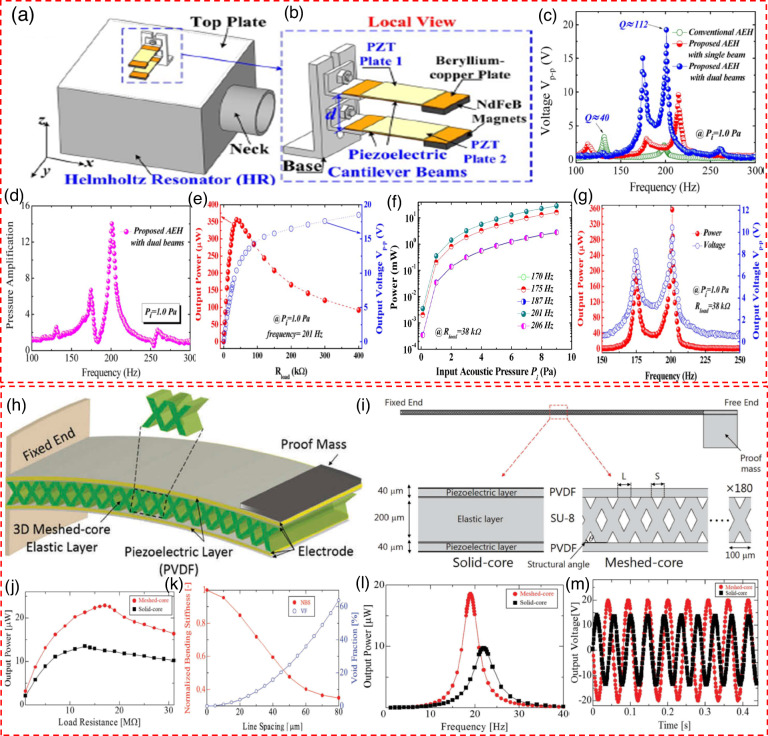
Utilizing cantilever structures for the piezoelectric nanogenerator to enhance acoustic energy harvesting performance. **a** Top view of the proposed energy harvesting design, which uses an HR with a PZT plate integrated into a cantilever to capture sound energy^73^. **b** local view of the piezoelectric cantilever beams^73^. **c** Voltage output as a function of frequency, comparing the performance of conventional AEHIs with the proposed AEHI design, featuring single and dual beams^73^. **d** Pressure boost for the device as a function of frequency^73^. **e** The resultant power and voltage vs various loads under 201 Hz and 94 dB^73^. **f** Produced power versus SPL input with multiple frequency values^73^. **g** Generated power and voltage as a function of frequency^73^. Copyright © 2014 AIP Publishing LLC. **h** Diagram of the designed Piezoelectric Vibration Energy Harvester (PVEH), featuring a 3D meshed-core elastic layer, piezoelectric PVDF layers, and electrodes forming a cantilever structure.^74^. **i** A FEM analysis of the meshed-core PVEH compared to the solid-core type, using a model with two piezoelectric layers and an elastic layer. The analysis examines the tip deflection and bending stiffness of both designs to evaluate the enhanced flexibility of the meshed-core structure^74^. **j** Output power comparison between meshed-core and solid-core designs^74^. **k** Volume void fraction and normalized bending stiffness plotted against the line spacing of the meshed-core structure^74^. **l** Power produced as a reflection of the frequency of vibrations^74^. **m** Voltage signal for meshed and solid cores^74^. Copyright © 2018 The Author(s). Published by Informa UK Limited, trading as Taylor & Francis Group

Yang et al.^73^ unveil an AEH setup that showcases high efficiency and the ability to operate across a broad frequency range, as illustrated in Fig. 7a. Their design features an HR equipped with a flexible top plate and complemented by two piezoelectric cantilever beams, detailed in Fig. 7b. This AEH, upon interaction with acoustic waves, triggers resonant cavity pressure within the HR, facilitated by its cavity and the flexible top plate. The induced vibrations on the top plate are converted into electrical energy via the dual piezoelectric cantilever beams, optimizing energy capture and minimizing losses through its high-Q beams and innovative design. The design achieves exceptional efficiency and broad frequency response when the dual beams and the flexible top plate of the HR resonate at the same frequencies, creating a robust coupling between the HR cavity, the vibrating plate, and the beams. This leads to significant oscillations in the cantilever beams, enhancing harvesting efficiency over a wide frequency spectrum. Figure 7c presents the output voltage versus frequency for three AEH configurations: the proposed dual-beam HR setup, an HR with a single beam, and a conventional HR with a piezoelectric composite diaphragm. The dual-beam configuration outperforms the conventional approach, yielding a maximum output voltage of 19.2 V at 201 Hz, ~6.4 times higher than the conventional setup. This enhancement is attributed to the dual beams’ high Q factor, which significantly reduces energy losses and boosts energy generation at resonance. The conventional AEH configuration’s dual peaks in output voltage result from mode coupling between the top plate and the HR cavity, with dominant frequencies linked to each component. The proposed dual-beam configuration shows a marked increase in vibration strength within the 170–206 Hz range, creating a 5.3–13.7 times pressure amplification than the conventional setup, as depicted in Fig. 7d, correlating with the output voltage peaks in Fig. 7c, indicating an acoustic-electrical coupling in the system. The relationship between output power and voltage against load resistance (R_load) at the resonant frequency of 201 Hz is shown in Fig. 7e, achieving a peak power output of 356 μW at an optimal R_load of 38 kΩ with an input SPL of 94 dB. This setup reaches a peak-to-peak voltage of 10.4 V at maximum power output. Further analysis, shown in Fig. 7f, explores the output power as a function of input acoustic pressure at various frequencies, demonstrating a substantial increase in output power with rising input pressure. At a frequency range of 170–206 Hz and an input pressure of 2.0 Pa (100 dB), the setup achieves maximum and minimum power outputs of 1.43 mW and 0.137 mW, respectively, highlighting its efficiency and potential for broad operational bandwidth. Comprehensive testing across different frequencies, maintaining an R_load of 38 kΩ and input pressure of 1.0 Pa, reveals that the AEH produces output power ranging from 34 to 356 μW and output voltages from 3.2 to 10.4 V over the frequency range of 170–206 Hz, as demonstrated in Fig. 7g. Tsukamoto et al.^74^ proposed meshed-core piezoelectric vibration energy harvester (PVEH) features a cantilever structure with electrodes, dual layers of piezoelectric material, and an interposed elastic layer made of thick-film photoresist with a 3D meshed-core structure, aimed at lowering the resonance frequency, as depicted in Fig. 7h. Traditional methods for reducing resonance frequency often involve adding a heavier proof mass, leading to increased device mass and volume, which limits practical applications. The meshed-core structure introduces a novel approach by incorporating a flexible layer within the piezoelectric array to circumvent these drawbacks. A comprehensive structural analysis conducted using FEM software COMSOL Multiphysics 5.2a contrasts the standard solid-core with the innovative meshed-core design (Fig. 7i). The output power comparison in Fig. 7j demonstrates the meshed-core’s superior performance, producing nearly double the output power at 20 µW compared to the solid-core. Figure 7k details the FEM analysis outcomes, showcasing the meshed-core structure’s ability to significantly reduce bending stiffness in the PVEH system, the graph plots the meshed-core line spacing against the normalized bending stiffness, indicating a stark reduction in stiffness compared to the solid-core design, thus validating the meshed-core’s efficacy. The resonance frequency and output power comparison, shown in Fig. 7l, reveals that the meshed-core PVEH resonates at 18.0 Hz, a substantial decrease from the 22.1 Hz resonance of the solid-core version. This 18.6% lower resonance frequency, coupled with a 74.5% increase in output power, underscores the benefits of reducing bending stiffness through the meshed-core structure. Additionally, Fig. 7m illustrates the output voltage waveform of the PVEH under resonance, highlighting the meshed- and solid-core variants’ performance. The meshed-core PVEH not only demonstrates a sinusoidal output voltage waveform in alignment with the input excitation but also achieves a 42.6% higher output voltage than its solid-core counterpart, reaching a peak output voltage of 20.4 V compared to 14.1 V for the solid-core.

#### Quarter wavelength-based PENG

The quarter-wavelength-based PENG is a distinctive arrangement that capitalizes on the resonance phenomena to optimize the efficiency of energy conversion. The device employs the principle of a quarter-wavelength resonator, enabling effective extraction of mechanical energy at certain frequencies. The fundamental configuration of a quarter-wavelength-based PENG comprises a layer of piezoelectric material that is positioned between two immovable terminations or reflecting barriers. When the device is subjected to mechanical vibrations or waves with the suitable frequency, these disturbances propagate along the piezoelectric layer, inducing areas of increased and decreased mechanical strain inside the material. The primary benefit of the quarter-wavelength-based PENG is in its capacity to attain a notable level of energy conversion efficiency. This is accomplished by aligning the resonant frequency of the PENG with the frequency of the external mechanical vibrations. The phenomenon of resonance serves to enhance the mechanical strain and, as a result, the electrical output, making it a very suitable option for the purpose of energy harvesting from targeted vibration sources.

**Fig. 8:**
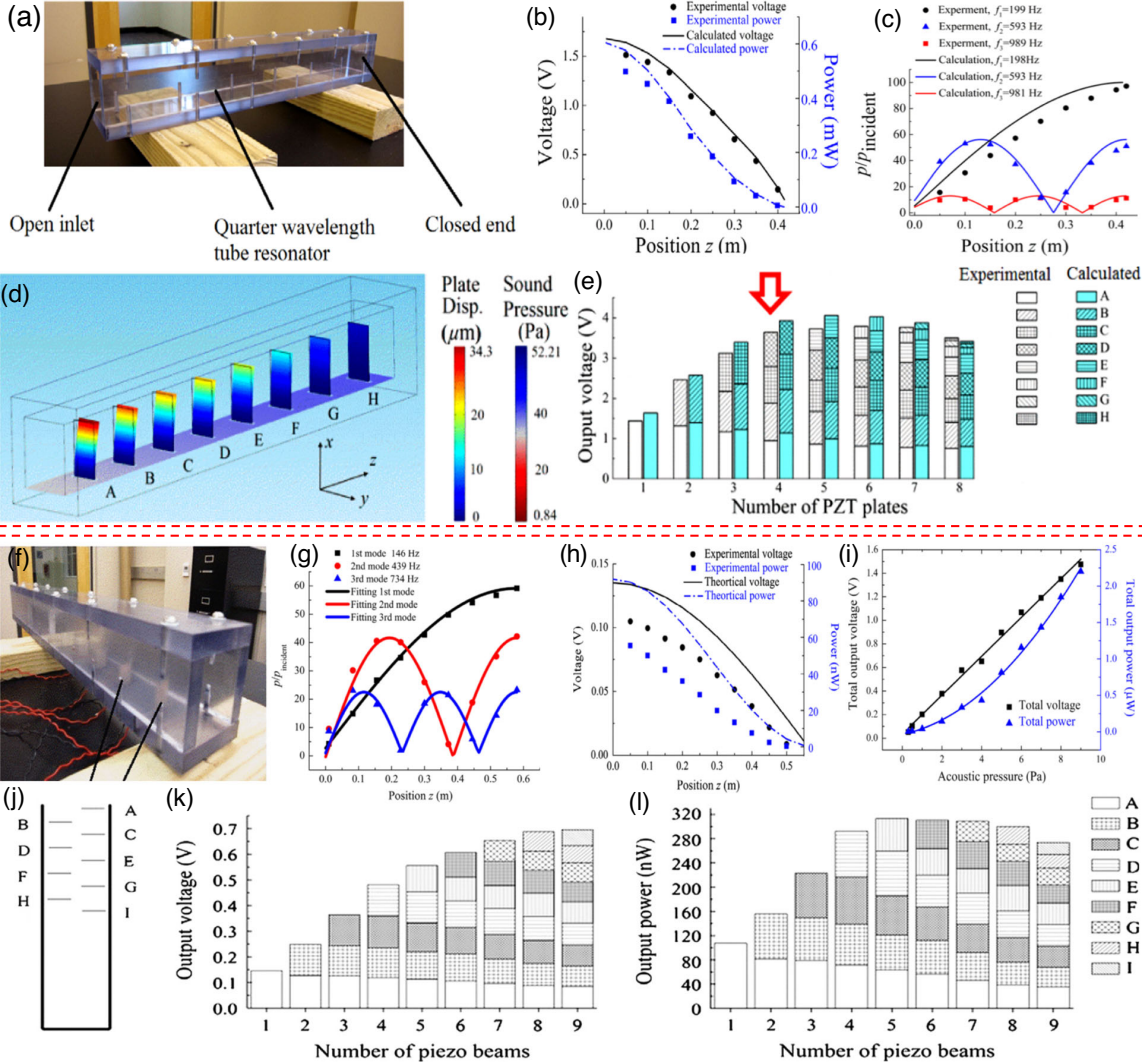
Optimizing acoustic energy harvesting with quarter wavelength tube resonator in piezoelectric nanogenerator. **a** Experimental setup featuring a quarter wavelength tube resonator and with an open and closed end^75^. **b** comparing the voltage and power produced by one plate from different locations throughout the tube between tested and calculated values^75^. **c** Pressure values that have been estimated and tested are normalized by the incoming acoustic pressure at the tube’s initial three eigenmodes^75^. **d** Illustration showing the calculated displacements caused by the acoustic wave of 8 piezo plates positioned within the tube^75^. **e** Effect of the number of piezo plates on the generated voltage^75^. Copyright © 2013 IOP Publishing Ltd. **f** Experimental setup showing the quarter wavelength tube^76^. **g** The values of the first three normalized eigenmodes of sound pressure within a straight tube are characterized by a rectangular inlet shape^76^. **h** One PVDF beam output voltage and power measurements at different locations experimentally and theoretically^76^. **i** The impact of incoming acoustic pressure on output voltage and power^76^. **j** Diagrams of zigzag arrangements with the beams in the tube’s first half and (**k**) the voltages and (**l**) power generated from these arrangements with varying numbers of beams^76^. Copyright © 2013 Elsevier Ltd. All rights reserved

Li et al.^75^ delved into harnessing low-frequency acoustic energy through a straight tube acoustic resonator equipped with PZT piezoelectric cantilever plates. By modifying the tube’s length, the researchers demonstrated that the frequency of energy harvesting could be effectively adjusted. The resonator, stimulated externally at its eigenfrequency, produced a resonant standing wave, with the PZT plates positioned perpendicular to the tube axis to convert these vibrations into electricity, as shown in Fig. 8a. Figure 8b details the energy harvesting performance with a piezoelectric plate placed at varying distances (z) from the tube’s entrance. Notably, a plate positioned 5 cm from the entrance yielded 1.639 V and 0.577 mW under a 196 Hz sound frequency. However, moving the plate closer to the tube’s closed-end decreased voltage and power, indicating reduced energy conversion efficiency with increased distance from the tube inlet. The effectiveness of this energy harvesting approach was further assessed through the amplification ratio (A)—the maximum pressure inside the tube to the incident sound pressure. Amplification ratios at various frequencies underscored the precision of the modeling process, with the first resonant mode (f1) showing the highest amplification and thus deemed most effective for energy harvesting, as depicted in Fig. 8c. To enhance output voltage and power, a setup with multiple piezoelectric plates within the tube resonator was tested, maintaining a constant SPL of 100 dB while varying the frequency. This setup, featuring up to eight plates, is illustrated in Fig. 8d, where plates are spaced 5 cm apart, starting from 5 cm at the tube inlet. Figure 8e plots the total output voltage against the number of plates installed, indicating an optimal arrangement at six plates for the highest voltage output of 3.79 V at a 193 Hz frequency. Additional plates beyond this optimal number resulted in reduced voltage output due to changes in acoustic resonance caused by extra plates near the tube’s closed end, suggesting a balance between maximizing energy capture and maintaining resonator efficiency. Li et al.^76^ proposed integrating a quarter-wavelength straight-tube acoustic resonator with PVDF piezoelectric cantilever beams to improve the efficiency of harvesting acoustic energy from low-frequency sound waves (Fig. 8f). In their study, the tube resonator was subjected to sound waves at 100 dB to identify its acoustic eigenmodes, with measurements of acoustic pressure taken along the tube length. Figure 8g showcases the first three normalized acoustic pressure eigenmodes along the tube, revealing eigenfrequencies of f1 = 146 Hz, f2 = 439 Hz, and f3 = 734 Hz, with resonant pressures peaking near the tube’s closed end. The amplification ratio, representing the highest pressure inside the tube relative to the incident sound pressure, highlighted the first eigenmode as having the highest amplification (59.1), with subsequent modes showing decreased ratios (42.2 for the second mode and 23.3 for the third). The experimental setup also involved positioning a single PVDF beam at varying locations within the tube to gauge the harvester’s response at the first eigenfrequency (f1 = 146 Hz). The findings, depicted in Fig. 8h, showed the highest output voltage (0.105 V) near the tube’s entrance, with voltage diminishing as the beam moved towards the closed end. the voltage and power outputs were analyzed in relation to incident acoustic pressure, as shown in Fig. 8i. At an incident pressure of 9 Pa (equivalent to an SPL of 110 dB). For the performance of the device, the voltage reached 1.48 V, predicting a power output of 2.2 μW and a power density of 0.11 μW/cm^2^ at this SPL level. The study further explored two tube configurations: a conventional straight design and a novel zigzag configuration shown in Fig. 8j. Output voltages and power for the zigzag configuration are detailed in Fig. 8k, l, illustrating the performance of piezoelectric beams at different tube positions under a 100 dB sound wave at 146 Hz. Implementing a zigzag setup with five PVDF beams placed from points A (*x* = 2.5 cm) to E (*x* = 12.5 cm).

#### Helmholtz resonator based PENG

The HR is a device used for the purpose of selectively attenuating or amplifying certain sound frequencies. The apparatus comprises a container with a narrow aperture, which is linked to a bigger compartment designed to hold a small amount of air^77^. The performance of the resonator is dependent upon several elements, including the volume and size of the chamber, the shape of the aperture, and the mass of the confined air. When a sound wave with a frequency proximate to the resonance frequency of the HR enters through the device, the enclosed air undergoes oscillations in a distinct manner, leading to the amplification of the input signal. HRs and mass-spring-damping systems are typical examples of basic harmonic oscillators in distinct domains (acoustics and mechanics, respectively)^78^. Resonance is seen in these systems, whereby oscillations occur with increased amplitude at distinct frequencies referred to as resonance frequencies. The efficiency of energy harvesting may be improved by successfully gathering and converting acoustic energy via the integration of an acoustic nanogenerator with HR. The adaptability of the resonator to certain frequency ranges enables it to effectively operate as a responsive device for collecting and amplifying acoustic energy. By adjusting the design of the resonator to align with the frequency of surrounding ambient sound, it is capable of effectively detecting, amplifying, and converting even minimal quantities of acoustic energy. This characteristic makes it very favorable for supplying energy to low-power devices in environments with limited noise tolerance^79^. The optimization of the system may be achieved by the integration of numerous resonators that are tuned to various frequencies, hence enabling broadband energy harvesting.

**Fig. 9:**
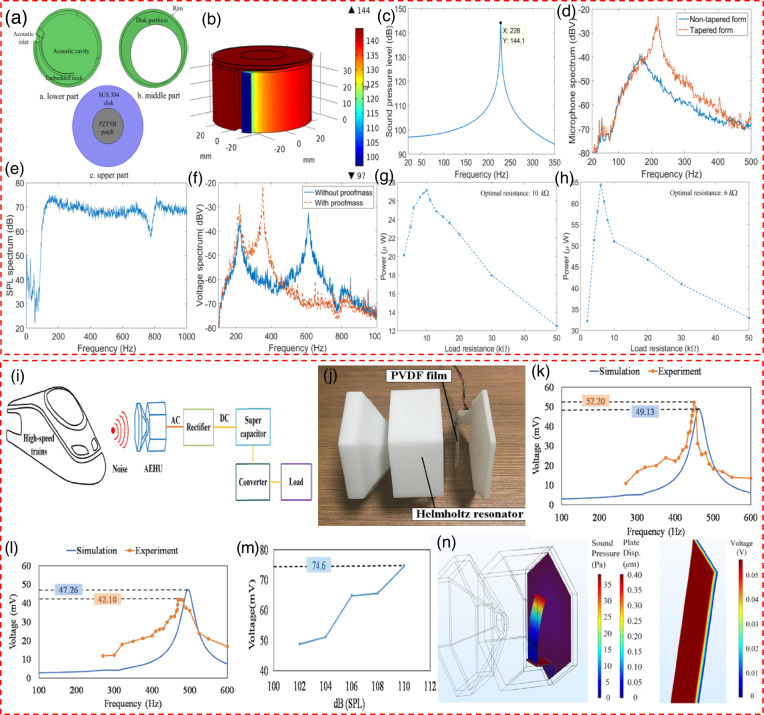
Utilizing the Helmholtz resonator to enhance input signal for acoustic energy harvesting using piezoelectric nanogenerator. **a** Top view of the Acoustic Energy Harvester (AEH) showing the cylindrical shaped HR with upper, middle, and lower parts, including the PZT piezoelectric plate positioned in the middle of the resonator^80^. **b** internal SPL distribution among the device^80^. **c** The SPL as a function of the frequency showing a huge jump close to the resonance frequency^80^. **d** Microphone-measured signal across a range of frequencies, showing comparisons between tapered and non-tapered designs^80^. **e** SPL spectrum of the loudspeaker’s frequency response^80^. **f** V_oc_ of the device with and without proof of mass^80^. Generated power against different load values (**g**) with a proof mass^80^ and (**h**) without proof mass^80^. Copyright © 2018 Author(s). All article content, except where otherwise noted, is licensed under a Creative Commons Attribution (CC BY). **i** Diagram of the setup for the Acoustic Energy Harvester for Noise Barrier (AEHNB), illustrating the conversion process from acoustic signals produced by high-speed trains to electrical energy, including rectification and storage components^81^. **j** Side view of the real acoustic energy harvesting setup, including the PVDF film and the HR^81^. **k** Comparison of simulated and experimental voltage outputs at different frequencies in Model One which is without noise cancellation^81^. **l** Voltage responses of the AEH in Model Two (with noise cancellation) across various frequencies, comparing experimental and simulation^81^. **m** Experimental device’s Voltage in Model Two at 447 Hz under Varied Sound Pressure Levels^81^. **n** Simulated displacement patterns of the device under acoustic excitation and the corresponding voltage generation, providing insight into the mechanical and electrical behavior of the system in Model Two^81^. Copyright © 2018 Elsevier Ltd. All rights reserved

Yuan et al.^80^ discuss a novel design for an AEH, which is based on the principles of a planar HR. This AEH is distinct from the traditional HR in that it has a more compact shape and a neck that has been purposefully built to help reduce acoustic viscous loss. There are three primary parts that go into making up the AEH system. The lowest portion of the system consists of an acoustic cavity that is surrounded by solid walls. Second, a disk partition that has an eccentric hole acts as the middle portion of the resonator. The last section of the AEH system is the top component, which is a metallic disk made of SUS 304. This disk is attached to the rim of the center part using screws. A PZT-5H piezoelectric patch, which is in the middle of this metallic disk (as seen in Fig. 9a). Figure 9b depicts the distribution of the SPL inside the proposed AEH structure at its resonant frequency. Along the neck that was immersed into the AEH, a gradual rise in SPL was observed, with the distribution of internal SPL being uniform at the resonance frequency. The computed peak internal SPL values are shown in Fig. 9c. When there is acoustic resonance present as a result of amplification, the internal SPL obviously goes up by a large amount; nevertheless, when there isn’t any resonance present, the SPL stays at a low level. The simulation, on the other hand, does not consider acoustic dampening, which may lead to an overestimation of the internal SPL value within the context of this basic numerical model. To conduct a head-to-head test against other configurations to validate the efficiency of the tapered neck arrangement and to illustrate the benefits it offers. The use of 3D printing processes resulted in the creation of two samples, one of which had a neck that was tapered while the other had a neck that was uniform. According to the findings of the tests, which are shown in Fig. 9d, the tapered shape resonator demonstrates improved sound pressure amplification capabilities while operating at the acoustic resonant frequency. In addition, the acoustic resonant frequency occurs at a larger value when the neck is tapered in comparison to the sample that does not have the neck tapered. In order to prepare the speaker for the measurement of the performance of the AEH, a band-limited version of noise with a frequency range of 100–1000 Hz was utilized as the excitation signal. As can be seen in Fig. 9e, this provided an illustration of the frequency response characteristics of the loudspeaker. This SPL spectrum reveals a wealth of information on the dynamic capabilities of the loudspeaker. For instance, the strength of the excitation was somewhat greater inside the frequency band that ranged from 100 to 200 Hz, as shown in Fig. 9e. This was the case in contrast to the higher frequency bands. In addition, a drop in the spectrum between 700 and 800 Hz suggested that the frequency region in question was only moderately stimulated. The research findings presented in Fig. 9f show two scenarios: one without an extra proof mass (solid line) and the other with a 9.5-gram proof mass (dotted line). Both scenarios exhibit two voltage peaks within the frequency range of 100–1000 Hz. The two voltage peaks are not due to inconsistent loudspeaker excitation but rather the result of acoustic resonance and mechanical resonance within the AEH system. The acoustic resonance, which is intrinsic to the AEH structure, has a resonance frequency of 221 Hz when no proof mass is present, matching the numerical forecast. With a proof mass, the acoustic resonance frequency is slightly lower at 220 Hz. The metallic disk’s mechanical resonance causes a second voltage peak to appear at frequencies higher than the first. Without a proof mass, this peak occurs at 611 Hz, and with the addition of a proof mass, the mechanical resonance decreases to 352 Hz due to mass-loading effects. The results, which are displayed in Fig. 9g, h, make it clearly evident that the incorporation of a proof mass resulted in a significant increase in the performance of the AEH system. When the proof mass was added, the greatest amount of power that could be collected grew to 27.2 μW during the acoustic resonance, and it climbed even further to 64.4 μW during the mechanical resonance. Wang et al.^81^ explored a cutting-edge noise reduction strategy for high-speed railways, employing a hybrid system that combines an HR with a PVDF film to simultaneously diminish noise pollution and harness acoustic energy for electricity generation. They developed an acoustic energy harvesting noise barrier prototype, with the electrical setup showcased in Fig. 9i. The design features a hexagonal prism cavity, utilizing a honeycomb structure to function as an HR, where the honeycomb’s outlet connects to the HR’s neck. This design enhances sound pressure around the resonator’s fundamental frequency when noise travels through the honeycomb structure and neck, as depicted in Fig. 9j. The study contrasts two models—one lacking a noise collection module and the other including it—to highlight the importance of incorporating a noise collection input module in the Acoustic Energy Harvesting Unit (AEHU)’s design. Figure 9k illustrates the discrepancies between actual and simulated results. Initial AEHU tests reveal that resonant sound pressure at 496 Hz induces the PVDF film to generate 47.26 mV and 0.48 μW, according to simulations. However, experiments yielded slightly lower values—42.10 mV and 0.38 μW at a reduced frequency of 468 Hz. Further simulations and experimental tests on a second model, integrating the noise collection module, demonstrate enhanced performance. Simulated outcomes predicted 49.13 mV and 0.52 μW at 464 Hz, while actual tests reported a higher voltage of 52.20 mV and power of 0.59 μW at 445 Hz as shown in Fig. 9l. This discrepancy indicates the prototype’s improved energy harvesting capabilities under experimental conditions. Figure 9m details the AEHU’s performance at various SPLs, recording a peak voltage of 74.6 mV and power output of 1.24 W at 110 dB SPL at 447 Hz, showcasing the significant improvement afforded by the first module. Figure 9n further demonstrates the AEHU’s simulated displacement and output voltages in the enhanced model, affirming the hybrid system’s potential for effective noise mitigation and energy harvesting on high-speed railroads.

Izhar et al.^82^ developed a wideband, three-degree-of-freedom AEH featuring an advanced HR with a chamber of conical shape and a bimorph piezoelectric composite plate. The experimental setup was further enhanced by including a cantilever beam composed of a brass base, a copper rod, and a steel plate alongside the piezoelectric plate, aiming to introduce an additional resonance frequency to expand the device’s frequency spectrum. In their analysis of the frequency response related to cavity pressure at different SPLs, the HR device with a cylindrical cavity showed a resonance peak at 1118 Hz. Conversely, the HR device with a conical cavity displayed a peak at 1538 Hz, signifying its resonance frequency. Notably, under a 130 dB SPL, the conical HR demonstrated higher internal cavity pressure (201 dB) compared to the cylindrical HR (194 dB). Utilizing COMSOL Multiphysics for simulation, the researchers observed pressure fluctuations within each HR device’s cavity at their respective resonance frequencies, under stimulation at 130 dB SPL. These simulations revealed a gradual increase in pressure from the orifice to the bottom along the longitudinal axis within the resonators. Specifically, the conical HR exhibited the highest peak SPL at 201 dB, underscoring its superior performance in pressure amplification.

### Special mechanism-based PENG

Piezoelectricity is a unique property of ferroelectric materials that may be distinguished by the structured molecular structure of the substance. They may generate an electric charge when subjected to mechanical stress or pressure because of this characteristic^83,84^. The random arrangement of the electric dipoles inherent in these materials in their natural state results in a balanced distribution of positive and negative charges throughout the material, and as a result, there is no net voltage difference across the material. The piezoelectric effect will occur once an external force or stress is applied to this arrangement, causing the material to distort. This mechanical stress will cause variations in the dipole density within its structure^85^. The piezoelectric effect is caused by the application of an external force or stress to a ferroelectric material, which causes the material to undergo deformation. This mechanical stress will lead to changes in the dipole density that is contained within its structure. Thus, the previously disorganized electric dipoles begin to reorganize and realign themselves in a particular direction^86^. This realignment separates positive and negative charges along the direction of the applied force, resulting in an electric polarization within the material. When the mechanical tension is released, the ferroelectric material returns to its original form and the reconfigured dipoles relax back to their original random configuration. This relaxing process creates an electric charge, which causes a voltage to be generated across the material. The amount of the applied force or stress is correlated to the magnitude of the voltage, allowing for accurate control and monitoring of the piezoelectric effect. A piezoelectric material is connected to a flexible or rigid surface to create the base of the PENG. To collect the electric charges created, electrodes are placed on opposing sides of the piezoelectric material. Initially, without any external force, the piezoelectric material lacks polarization. However, when external pressure is applied, the material compresses, causing a separation of charges and the generation of an electric potential difference across the electrodes. By connecting the electrodes to external conductive wires, charges can flow between them, effectively converting mechanical energy into electrical energy. When pressure is continuously applied, the dipole density within the material reaches its maximum. Upon releasing the pressure, electrons flow back to restore balance in the induced charge. As a result, a continuous reciprocal force applied to the PENG establishes a steady-state sinusoidal current in an external circuit.

The operating mechanism of the MWCNTs/PVDF-TrFE/PDMS (MCPP) ANG may be clarified as follows^60^: The periodic oscillation of a material, caused by a sinusoidal sound wave, is followed by a variation in air pressure on each side of the material. This pressure difference induces a cyclic deformation of the material and creates strain on its surface. Consequently, the material undergoes a change in its dipole moment, leading to the generation of a piezoelectric output. Concurrently, as a result of the phenomenon of diffraction and scattering of sound waves inside the porous material, the unequal transmission of these waves between the walls of the pores and the pores themselves would give rise to internal friction and the production of triboelectric charges. Furthermore, the transfer of triboelectric charges is seen during the cyclic contact and separation of the embedded PDMS tympanum from the pore wall due to the contrasting triboelectric charging affinities and polarity between them. In addition, the inclusion of a suitable quantity of MWCNTs has been seen to enhance the propagation of triboelectric and piezoelectric charges by facilitating the establishment of conductive networks. The transfer process may be shown in the following manner: In the condition of initial equilibrium, there is an absence of charge transfer. The surface charges of the MCPP composite material undergo changes because of the periodic vibration and strain generated by sound waves, leading to the induction of charge transfer in the external circuit. Moreover, the flow of external circuit charges may be attributed to the transport of free electrons from the pore wall of PVDF-TrFE to the tympanum of PDMS, which is facilitated by the increased electronegativity of PDMS compared to PVDF-TrFE. The dissipation of the sound wave leads to the restoration of strain, which induces a reversal in the direction of current flow, so generating a reverse signal. Hence, the MCPP ANG has the capability to produce amplified alternating current (AC) when subjected to sinusoidal acoustic stimulation, owing to the combined influence of piezoelectricity and triboelectricity^65^. For the GaN-ZnO nanogenerator mechanism, the initial state, when there are no external stimuli applied, the device remains in a quiescent state, producing no piezoelectric potential. However, when an external compressive force is applied to the nanogenerator, it results in the distortion of the ZnO and PAN layers, an event that triggers the generation of a piezoelectric potential. This generated potential is a consequence of the strain exerted on the material and the resulting structural alterations. This structural change leads to the induction of electrons which are displaced across the external circuit. The electrons eventually accumulate at the intersection between the base electrode and the ZnO nanorods, which have a positive charge due to the induced potential. This congregation of electrons leads to the generation of a positive current pulse. The process is made more complex by the presence of free electrons around the nanorods. These electrons act as a protective shield, effectively “screening” the positive potential generated during the compression process. As these screening electrons move from the upper electrode and intrude into the ZnO region through the Ohmic contact, it causes a reduction in the overall positive potential. During the decompression or release phase, the PAN film reverts to its original form, leading to the generation of a counter-electric field. Consequently, the diminished positive potential at the bottom is insufficient to propel the accumulated electrons from the base electrode back up to the top electrode. This results in a situation where no pulse signal is detected during the decompression phase. Instead, a steady, DC output is generated. In essence, the PENG has the capability to convert mechanical energy, derived from both compression and decompression cycles, into electrical energy. This conversion process involves the generation and effective manipulation of the piezoelectric potential. The ultimate result of this complex process is the generation of a DC signal, demonstrating a successful conversion of mechanical energy into electrical energy. The AC output mode seen in the nanogenerator may be explained by the establishment of Schottky connections between the nanowires and the electrode, which serves as a capacitive element^70^. When a sound wave generates a negative piezoelectric potential, it results in an increase in the conduction band and Fermi level on the upper surface of the nanowires. As a result, the movement of electrons is initiated from the upper electrode towards the lower side through an external circuit, resulting in the creation of a positive potential around the upper PdAu electrode. The Schottky contact serves to impede the flow of electrons over the interface, so preserving the produced potential. Nevertheless, when the external force exerted on the upper PES substrate is temporarily relieved during the rarefaction phase of the sound wave, the nanowires experience a release of compressive strain. Therefore, the dissipation of the piezoelectric potential occurs, leading to the reflow of electrons inside the external circuit until equilibrium is reached and the positive potential around the top electrode is neutralized. The aforementioned cyclical phenomena involving the development and dissipation of potential occur in a repetitive manner, ultimately leading to the production of AC voltage pulses through the nanogenerator. Therefore, the nanogenerator efficiently utilizes the piezoelectric potential generated by the sound wave, transforming it into an oscillating AC output.

The discussion on the special mechanisms of PENGs enriches our understanding of how advanced materials and structural designs can be leveraged to optimize the conversion of mechanical energy into electrical power. Highlighting the intricate interplay between piezoelectric and triboelectric effects, this section illuminates the innovative strategies employed to enhance energy harvesting efficiency. Through the exploration of mechanisms such as the dynamic alignment of dipole moments under mechanical stress and the sophisticated integration of materials like MWCNTs and PDMS, these special mechanism-based PENGs underscore the potential for significant advancements in energy conversion technologies. By delving into the complexities of how external forces induce changes in dipole density and piezoelectric potential, the text illustrates the critical role of ferroelectric materials’ structured molecular arrangements in generating electrical charges. Moreover, the nuanced explanation of how the combined piezoelectric and triboelectric effects contribute to the generation of AC.

### Frequency band

As seen in the provided papers above, the frequency range for acoustic energy harvesting is very wide, spanning almost from 1 Hz up to 8000 Hz. The material of the nanogenerator plays a crucial role in determining its frequency sensitivity and efficiency. Different piezoelectric materials, such as PVDF, PZT, and PVDF-TrFE, exhibit varying sensitivities at different frequencies. For example, PVDF is often used in low to medium-frequency applications due to its flexibility and high piezoelectric constant in the β-phase. Structural design also significantly impacts the operational frequency range. Configurations like cantilever structures, aerogels, or nanofiber devices affect the resonant frequency and, consequently, the efficiency across different frequency bands. The structural configuration of nanogenerators plays a pivotal role in determining their operational frequency range and overall efficiency. Designs incorporating cantilever structures, aerogels, and nanofiber devices are tailored to optimize resonant frequencies, thereby enhancing energy capture efficiency. For instance, cantilever-based nanogenerators are adept at harnessing low-frequency vibrations, as demonstrated by Tsukamoto et al.^74^ whose device operates effectively between 1 and 22 Hz. On the other hand, Izhar et al.^82^ developed a nanogenerator designed for higher frequencies, reaching up to 2000 Hz. These design choices underscore the importance of aligning the structural attributes of nanogenerators with their intended frequency applications. The mid-range frequencies, spanning from 100 to 500 Hz, are often the sweet spot for AEHs. This range strikes a balance between providing sufficient cycles of contact separation and maintaining adequate charge transfer, essential for efficient energy generation. Additionally, this frequency band aligns well with common environmental and urban noise frequencies, ensuring a consistent and reliable energy source. At higher frequencies, the rapid separation of contact points can lead to inadequate charge transfer, diminishing energy capture efficiency. Conversely, lower frequencies may not offer enough cycles of contact separation to generate significant energy. Advanced structural designs, such as those incorporating HRs and quarter-wavelength tubes, are crucial in enhancing resonance at specific frequencies, thus improving energy capture efficiency. HRs are particularly effective in amplifying low-frequency sound waves, making them ideal for environments with predominant low-frequency noise. Quarter-wavelength resonators, conversely, excel at higher frequencies, thereby broadening the operational frequency range when integrated with other structural elements. The integration of composite fibers and multi-layered structures significantly broadens the frequency response range of nanogenerators. Electrospun nanofibers, for instance, can be precisely engineered to exhibit specific dimensions and mechanical properties, resulting in devices that are highly sensitive across a wide range of frequencies. Furthermore, combining different piezoelectric materials within a single device can enhance its performance across various frequency bands, offering a versatile and efficient energy-harvesting solution. Environmental factors, including temperature and humidity, also impact the frequency response of nanogenerators. Stability across varying environmental conditions is crucial for maintaining consistent performance. Research into hybrid materials and surface treatments that mitigate environmental effects is showing promising results, indicating potential improvements in stability and efficiency.

### Applications

**Fig. 10:**
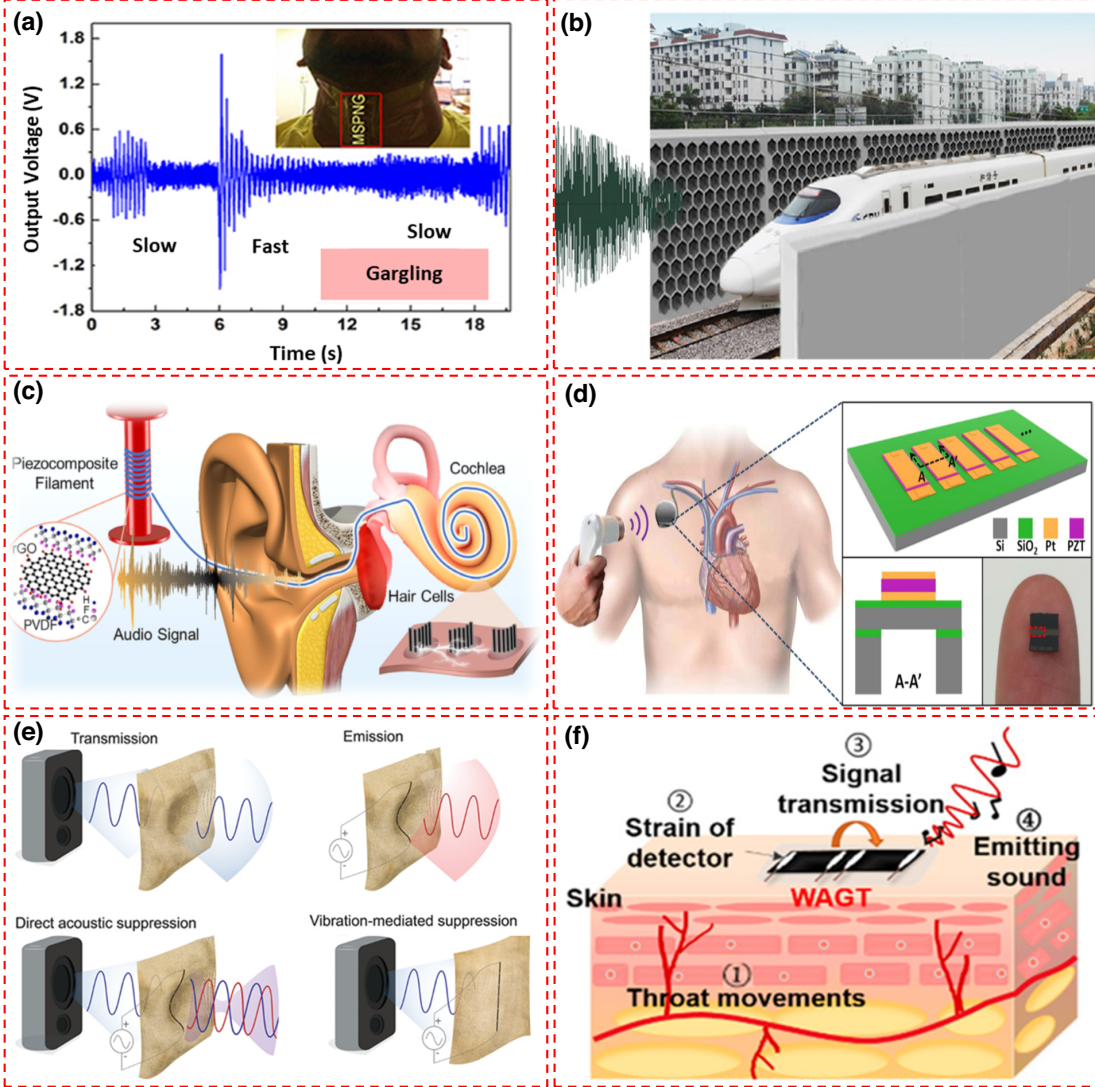
Applications of Acoustic Piezoelectric Nanogenerators. **a** Throat-attached nanogenerator used for self-powered wearable devices, capable of detecting vibrations from vocal activities like speaking or gargling, enabling real-time monitoring^57^. Copyrights © 2019 Elsevier Ltd, all rights reserved. **b** Noise reduction and energy harvesting in high-speed railways^81^. Copyright 2018 Elsevier Ltd. All rights reserved. **c** Nanostructured piezoelectric filaments for next-generation cochlear implants^88^. Copyright © 2016, The Author(s), all rights reserved. **d** Ultrasonic activation of PTFE particles for sonodynamic cancer therapy^87^. Copyright © 2024 Wiley & Sons, all rights reserved. **e** Multifunctional fabrics engineered for both sound emission and suppression, offering innovative solutions for noise control and active sound management^89^. Copyright © 2024 Wiley-VCH GmbH, all rights reserved. **f** Wearable artificial graphene throat for assistive communication, designed to detect throat movements and convert them into sound signals for improved communication capabilities^90^. Copyright © 2019 American Chemical Society, all rights reserved

Acoustic Piezoelectric Nanogenerators (A-PENGs) exhibit a wide range of applications across various fields due to their exceptional ability to convert acoustic energy into electrical power. One prominent application is in wearable technology. For instance, Karan et al.^57^ developed a throat-attached nanogenerator that utilizes vibrations from vocal cords to generate electrical power (Fig. 10a), enabling the development of self-powered wearable devices that do not require conventional batteries, significantly enhancing user convenience and device longevity. In urban infrastructure, A-PENGs are used to capture acoustic energy from environmental noise, such as traffic or industrial machinery, to power sensors and IoT devices. Wang et al.^81^ explored a cutting-edge noise reduction strategy for high-speed railways, employing a hybrid system that combines an HR with a PVDF film to simultaneously diminish noise pollution and harness acoustic energy for electricity generation. This application promotes energy sustainability and helps monitor and manage urban environments more effectively. Healthcare is another critical area where A-PENGs are making a significant impact (Fig. 10b). Additionally, Wang et al.^87^ proposed the ultrasonic activation of poly(tetrafluoroethylene) (PTFE) particles for biomedical applications, particularly in sonodynamic therapy for cancer treatment (Fig. 10c). This innovative approach involves using ultrasound waves to activate PTFE particles, which then generate reactive oxygen species (ROS) such as hydroxyl radicals (·OH), superoxide (·O2−), and singlet oxygen (1O2). These ROS are highly effective in inducing cytotoxic effects on tumor cells, offering a targeted and minimally invasive approach to cancer therapy. The biocompatibility and chemical stability of PTFE make it an ideal candidate for this application, as it can safely interact with biological tissues without causing adverse effects. Mokhatri et al.^88^ proposed a self-powered nanostructured piezoelectric filaments that demonstrated their potential application in next-generation cochlear implants. These flexible filaments, made from PVDF, PVDF/BT, and PVDF/rGO, have shown promising results in converting sound vibrations into electrical signals, mimicking the natural function of cochlear hair cells. The PVDF/rGO filament, in particular, displayed high sensitivity and the ability to generate electrical output in response to sound stimuli, making it a viable candidate for use in cochlear implants (Fig. 10d). Yang et al.^89^ extended these applications by integrating piezoelectric fiber actuators with traditional fabrics, such as silk and cotton, to develop multifunctional fabrics capable of sound emission and active sound suppression. These fabrics can emit sound waves up to 70 dB and reduce noise levels by up to 37 dB through direct acoustic interference. Additionally, they demonstrated vibration-mediated suppression, achieving a 75% reduction in transmitted sound by suppressing fabric vibrations. These advancements offer practical solutions for noise control in various environments, including architecture and transportation, and highlight the potential of integrating piezoelectric elements with everyday materials (Fig. 10e). Moreover, Wei et al.^90^ introduced a novel wearable skin-like ultrasensitive artificial graphene throat (WAGT), which integrates both sound/motion detection and sound emission capabilities. The WAGT leverages the high sensitivity and excellent sound-emitting properties of graphene, allowing it to detect subtle throat movements and transform them into audible sounds. This technology provides a significant breakthrough for mute individuals, enabling them to “speak” by converting mechanical signals from throat movements into electrical signals and subsequently into sound. The dual-mode system, comprising sound detection and emission, allows real-time transformation of throat movements into corresponding sounds, offering a practical solution for assistive communication. The device’s thin, flexible, and skin-like structure ensures comfort and ease of wear, making it suitable for long-term use in healthcare and wearable technology (Fig. 10f).

### Limitations of the PENG

To counteract these consequences, continuing research aims to generate more durable materials and protective coatings that can withstand extreme environmental conditions while maintaining performance over long durations. While PENGs offer promising potential for energy harvesting, they face several significant challenges that could impact their long-term viability and performance. Two of the most critical issues are material degradation and efficiency, which are essential to address to advance PENG technology and its practical applications. One major challenge with PENGs is the degradation of piezoelectric materials over time. Materials such as PVDF and PZT, which are known for their high piezoelectric coefficients, tend to experience mechanical fatigue when subjected to continuous mechanical stress. This fatigue can lead to the development of microcracks and a gradual weakening of structural integrity. Environmental factors like temperature fluctuations, humidity, and chemical exposure can accelerate this degradation process. As these materials deteriorate, their ability to convert mechanical energy into electrical energy diminishes, reducing the efficiency and reliability of PENG devices. This degradation is particularly problematic for applications that require long-term, maintenance-free operation in harsh environments. Efficiency is another significant barrier to the widespread use of PENGs. Typically, PENGs produce a low electrical output, which may not be sufficient to power most modern devices directly. The efficiency of energy conversion in PENGs depends heavily on the alignment between the environmental vibration frequencies and the device’s resonance frequency. If these frequencies do not match, energy harvesting becomes inefficient, as maximum efficiency is achieved only at resonance. Additionally, electrical losses, such as dielectric losses within the piezoelectric material and resistive losses in the circuit, further reduce the amount of usable energy. These losses can significantly impact the overall performance of PENGs, making it challenging to generate the energy output needed for practical applications. To tackle these issues, ongoing research is focused on developing more durable materials and protective coatings that can withstand extreme environmental conditions while maintaining performance over long durations. Innovations in material science aim to enhance the mechanical and chemical stability of piezoelectric materials, thereby extending their operational lifespan. For example, incorporating nanocomposites and advanced polymer matrices can improve the resilience of piezoelectric materials against mechanical fatigue and environmental degradation. Protective coatings and encapsulation techniques are also being explored to shield PENGs from harsh environmental conditions, such as moisture and corrosive chemicals, which can accelerate material degradation. Efforts are also being made to improve the efficiency of PENGs by optimizing their design and materials. Advanced fabrication techniques, such as electrospinning and 3D printing, enable the creation of nanostructured piezoelectric materials with enhanced properties. These techniques can improve the alignment of piezoelectric domains, increasing the overall efficiency of energy conversion. Additionally, integrating multi-layered and composite structures can enhance the energy output of PENGs by maximizing the effective surface area and improving mechanical coupling. Research is also focused on developing adaptive and tunable PENGs that can adjust their resonance frequency to match the frequency of environmental vibrations. This approach can significantly improve energy harvesting efficiency by ensuring optimal resonance under varying conditions. The use of advanced signal processing and power management techniques can also help in maximizing the harvested energy and reducing electrical losses. Addressing these limitations through the development of advanced materials, protective coatings, and optimized designs is crucial for the future of PENG technology. Researchers are striving to create more reliable, efficient, and durable PENGs that can provide sustainable energy solutions for various applications.

### Future scope of acoustic energy harvesting

**Fig. 11:**
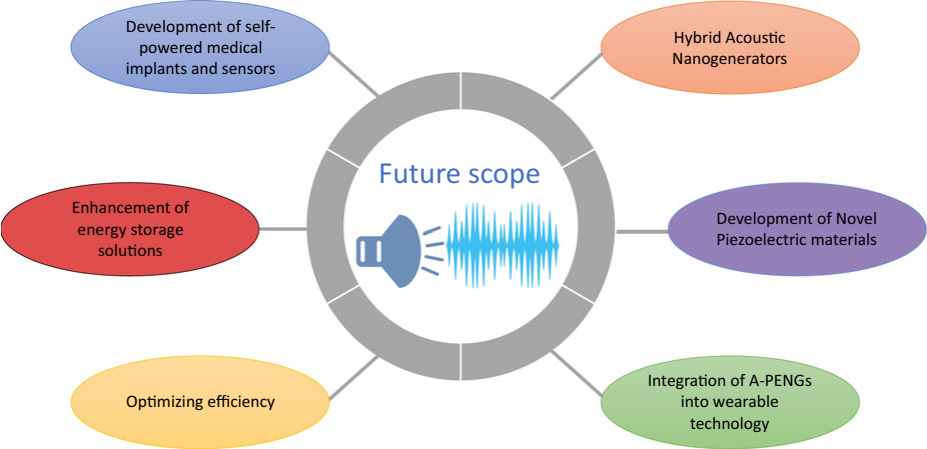
Future perspectives of acoutic energy harvesting. A schematic showing the future scope perspective for the A-PENG

Focusing on future advancements, the push towards refining the performance of PENG is evident through the investigation of groundbreaking materials. The pursuit of enhanced PENG efficiency is closely tied to utilizing novel piezoelectric materials known for their superior piezoelectric coefficients, which are also non-toxic and environmentally friendly. Optimizing energy transfer efficiency is a key goal, achievable by fine-tuning nanostructures within these materials. This involves alterations to surface morphology and incorporating hierarchical or asymmetrical designs to expand the contact surface area for improved energy capture. The potential applications of PENGs are extensive, ranging from wearable technology, where they can be integrated into items like clothing and watches to harvest energy from human movements or ambient sounds to urban infrastructure, where they can utilize acoustic energy from various sources for powering sensors and IoT devices^91,92^. Developing self-powered medical implants and sensors that utilize energy from body movements or acoustic waves holds significant promise in healthcare. Multiple applications and ideas were proposed. Mokhatri et al.^88^ proposed a self-powered nanogenerator, demonstrating their potential application in next-generation cochlear implants. However, realizing the full potential of PENGs involves overcoming challenges such as enhancing energy conversion efficiency, ensuring long-term stability under mechanical stress, addressing the economic and scalability aspects of production, and improving energy storage solutions. Nevertheless, to fully realize the potential of PENGs, several challenges need to be overcome. These include enhancing the energy conversion efficiency and ensuring the nanogenerators’ long-term durability against mechanical stress. Economic considerations, such as the cost-effectiveness and scalability of manufacturing processes, also play a critical role in the commercial success of these technologies. Moreover, improving the efficiency of energy storage solutions remains a daunting challenge. Although incorporating supercapacitors or batteries with these nanogenerators is feasible, there is considerable room for improvement in their storage capacity and charging capabilities. Lastly, the incorporation of hybrid tribo-piezoelectric (T-P) nanogenerators into the realm of acoustic energy harvesting has presented novel opportunities for enhancing energy conversion efficiency^93,94^. Integrating triboelectric and piezoelectric phenomena within a singular apparatus capitalizes on each mechanism’s distinct benefits, leading to enhanced capabilities for energy generation^95,96^. The hybrid T-P nanogenerators exhibit enhanced charge generation and collection efficiency^97^, allowing for more effective harvesting of acoustic vibrations. One of the examples of the hybrid nanogenerator is the work by Yu et al.^60^ that integrates triboelectric and piezoelectric principles into a single generator, enabling efficient capture of sound energy. Despite these hurdles, the strides made in enhancing the capabilities for energy generation through innovative material use and structural optimization provide a solid groundwork for future exploration and application in the field. A full schematic for the Future scope is shown in Fig. 11.

## Conclusion

This paper delves into the recent advancements in PENGs for acoustic energy harvesting, highlighting the significant potential of these devices in converting ambient sound into electrical energy. This paper explores the diverse applications, structural designs, and materials used in PENG technologies, highlighting their potential to efficiently convert mechanical energy into electrical energy through the piezoelectric effect. The study delves into various structural configurations, such as cantilever beams, HRs, and quarter-wavelength resonators, each offering unique advantages in optimizing energy conversion, summarized in Table 1. The paper also discusses the critical role of material selection, including natural and synthetic piezoelectric materials, in enhancing device performance.^84,98^. The use of piezoelectric materials in the design of nanogenerators, such as PVDF and ZnO^99^, has resulted in significant advances in this sector. PVDF exhibits different phases, the most common being the “A” and “B” phases. Typically, PVDF is predominantly found in the “a” phase. The “B” phase represents a specific molecular configuration within the PVDF material. When PVDF undergoes a transition into the “B” phase, it experiences an enhancement in its piezoelectric properties^53,100^. To improve PVDF’s piezoelectric properties, scientists have explored incorporating additional materials. For instance, Alam et al. conducted research involving the addition of TiO2 to PVDF, resulting in a 16% higher content of the β-phase, as reported in their paper^59^. Furthermore, Karan et al. introduced a bio-inspired VB2 into PVDF, leading to an overall enhancement in performance^57^. The paper also examines the integration of PENGs in wearable technology, urban infrastructure, and healthcare devices, offering a glimpse into the future of self-powered systems. Challenges such as energy conversion efficiency, long-term durability, and economic viability are addressed, with hybrid tribe-PENGs emerging as promising solutions for boosting efficiency. This comprehensive analysis underscores the pivotal role of PENGs in advancing renewable energy solutions and sets the stage for future research and development in acoustic energy harvesting. The sector has turned to specialized solutions like quarter-wavelength tubes and HRs to harness energy from low-intensity sound environments. These innovations excel in capturing and enhancing specific sound frequencies, offering a tailored approach to acoustic energy conversion. Their introduction is a significant stride towards boosting the efficiency of energy harvesting systems^101^. Integrating machine learning with acoustic nanogenerators has opened new avenues in monitoring and understanding complex acoustic patterns. The forward momentum in acoustic energy harvesting and the evolution of PENG technologies heralds a promising horizon for sustainable energy solutions. This trajectory is set to redefine our reliance on sound as a primary energy source, steering us towards a more sustainable and eco-conscious future. By embracing the nuanced capabilities of sound and refining its transformation into electrical energy, we stand on the brink of a paradigm shift in energy generation and utilization, paving the way for a sustainable and environmentally harmonious world^102,103^.

**Table 1:** Comparison table of Key Parameters for A-PENGs

No.	A-PENG	Maximum power (mW/m^2^)	V_oc_	I_sc_	db range	Frequency range (Hz)	size Cm^2^	Application	Practicality	Preparation difficulty	Ref
1	Fe/(MWCNTs/PVDF-TrFE/PDMS)/Fe	11.62 (Under115 db and 150 Hz @ 20 MΩ)	34.4 V	1.74 μA	95–115	110–400		broad sound frequency spectrum energy harvesting	Versatile for various applications	Moderate, requires precise material synthesis	^60^
2	Al/PVDF/Al		5.57 mV	15.6 nA	80–120	40–280		wearable Acoustic sensors	Easy integration into devices	Low, straightforward electrospinning	^61^
3	Conductive fabric/(CdI2/ 1,5- (NAP) /PVDF)/Conductive fabric	3.906 (Under110 db and 120 Hz @ 1 MΩ)	6 V	4 μA	60–110	20–330	16	acoustoelectric conversion and human motion detection	Good for flexible electronics	High, involves complex synthesis	^56^
4	(Ni–Cu) /PVDF-Mg/ (Ni–Cu)	(Under120 db and 130 Hz)	3 V		80–120	130	7.2	pressure detectors, self-powered microphones, energy harvesters	Highly durable	Moderate, involves precise alignment	^62^
5	Au/PAN/Au	175.3 (Under117 db and 230 Hz @ 4 MΩ)	58 V	12 μA	60–120	100–500	12	noise-harvesting devices	Applicable for large areas	Low, standard electrospinning technique	^64^
6	Ag/(BZT-BCT)/Ag	-	1.8 V	0.67 μA	79–104	50–400	4	a self-powered sound sensor	Easy to fabricate	Low, common composite fabrication	^68^
7	(Ni–Cu) /PVDF-TiO2/ (Ni–Cu)	17.4 (Under90 db @ 0.7 MΩ)	17.5 V	24 μA	40–90	-	60	self-powered devices.	High biocompatibility	Moderate, requires doping and sintering	^59^
8	Cu/(PVDF-VB2) /Cu	-	0.53 V	-	84	-	-	harvesting of multiple energy types.	Environmentally friendly	Moderate, involves vitamin incorporation	^57^
9	(Ni-Cu) /(Ce3 + -PVDF-Graphene) / (Ni-Cu)		3 V		88	62–110		pressure sensors	Enhances electrical properties	High, involves multiple material integration	^104^
10	Au/(PAN-ZnO) /(Cu-ZnO)		0.6 V	2.5 μA		200	2.25	self-powered electronics	Robust and stable	Moderate, layer deposition required	^65^
11	Cu/(PLLA-VB2)/Cu		0.4 mV			1–27,000		wearable devices, human-machine control systems	Biodegradable	Moderate, vitamin integration	^66^
12	Cu/(PVDF-ZnO)/Cu	2 (Under116 db and 140 Hz @ 0.7 MΩ)	1.12 V	1.6 μA	95–120	0–2000	12	noise energy collection	Enhanced piezoelectricity	Low, simple composite fabrication	^63^
13	Au/(Cellulose-SbSI)/Au	41.5 nW/cm^3^ (Under95 db and 175 Hz @ 11 MΩ)	24 mV	20 nA/cm^2^	95	100–250		mechanical and acoustic energy harvesting	Flexible and lightweight	High, nanowire growth on substrate	^105^
14	Al/PVDF/Al		0.7781 1 V		100	50–300		fine-tuning	High surface area	Low, standard electrospinning	^69^
15	(PdAu- PES)/(ZnO-GaN) /sapphire		50 mV		100	100		Energy harvesting	Suitable for nanodevices	High, precise nanowire alignment	^70^
16	bimorph piezoelectric plate	16.32 μW/cm^3^ (Under 130 db and 1501 Hz @ 1 KΩ)	461 mV		75–130	1000–2500	13.1 cm^3^	wide frequency range energy harvesting	Adaptable for various applications	Moderate, requires precise assembly	^82^
17	PZT-5H plate	64.4 μW (Under 100 db and 341 Hz)			100	100–1000		lower frequencies energy harvesting	Compact and efficient	Moderate, precise design and fabrication	^80^
18	PVDF film	0.38 μW (Under 100 db and 468 Hz)	42.1 mV		100	100–600		high-speed railway noise reduction	Suitable for structural integration	Low, standard film deposition	^81^
19	Au/P(VDF-TrFE) /Au	306.5 μW/cm3 (Under 100 db and 468 Hz @ 0.47 MΩ)	14.5 V	28.5 μA	60–120	100–2000		Acoustic energy harvesting	High output for small devices	High, complex material synthesis	^58^
20	PZT plate	0.137–1.43 mW			100	170–206		noise canceling	Wide application range	High, broadband design complexity	^73^
21	PZT plates	6350	15.6 V		110	180–205		low-frequency acoustic energy harvesting	Good for environmental noise	Moderate, precise tuning required	^75^
22	PZT-5H	7.3 μW (Under 100 db, 183 Hz and 11 KΩ)			100	183		low-frequency noise suppression	Compact and effective	Moderate, involves 3D printing	^106^
23	PVDF	1.1 Under 110 db	1.48 V		0–110	146–734	20	enhancing energy harvesting efficiency	Efficient energy conversion	Moderate, requires beam alignment	^76^
24	Ag-PZT-Ag	429 μW (Under 110 db, 554.5 Hz and 4.4 KΩ)	3.89 v		110	4000–7000		Noise cancellation, acoustic filtering,	High efficiency	High, involves precise coupling design	^71^
25	Al-PVDF-Al	24.6 μW (Under 18.7 Hz and 17 MΩ)	14.4 V			1–40		Wireless application sensors	High adaptability	High, involves complex core fabrication	^74^
